# Intrinsic functional and structural network organization in the macaque insula

**DOI:** 10.1162/imag_a_00261

**Published:** 2024-08-14

**Authors:** Joey A. Charbonneau, Erika P. Raven, Yuta Katsumi, Anthony C. Santistevan, Christopher Taylor, Eliza Bliss-Moreau

**Affiliations:** Neuroscience Graduate Program, University of California Davis, Davis, CA, United States; California National Primate Research Center, University of California Davis, Davis, CA, United States; Center for Biomedical Imaging, Department of Radiology, New York University Grossman School of Medicine, New York, NY, United States; Department of Neurology, Massachusetts General Hospital and Harvard Medical School, Boston, MA, United States; Department of Psychology, University of California Davis, Davis, CA, United States

**Keywords:** insula, interoception, functional connectivity, diffusion, monkey, comparative

## Abstract

In recent decades,*in vivo*magnetic resonance imaging (MRI) studies have provided previously inaccessible insights into the structure and function of healthy and pathological human brains in the laboratory and the clinic. However, the correlational nature of this work and relatively low resolution mean that ground truth neuroanatomical studies and causal manipulations of neural circuitry must still occur in animal models offering greater tractability and higher resolution, rendering a scale and species gap in translation. Here, we bridge this gap with a detailed, multimodal investigation of the macaque insula*in vivo*. Using both functional and diffusion MRI—tools available for use in humans—we demonstrate a neural architecture in the macaque insula with clear correspondence to prior*in vivo*MRI findings in humans and postmortem cytoarchitectural and tract-tracing studies in monkeys. Results converged across analysis methods and imaging modalities, supporting the translational potential of the macaque model.

## 1 Introduction

One of the major challenges of modern translational neuroscience is that the scale at which we carry out mechanistic work in nonhuman animals (e.g., manipulation or measurement of synapses, cells, and cytoarchitectonic regions) and the scale at which we measure neural structures and functions in humans (e.g.,*in vivo*neuroimaging) differ by many orders of magnitude. As a result of this difference in scale, virtually all*in vivo*neuroimaging studies report on features (e.g., volume, function, macrostructure) of brain areas that merge many cytoarchitecturally distinct subregions. Moving between levels of analysis is critically important as new treatments and interventions require understanding mechanisms which occur at the microscale, but tools available to monitor clinical populations—including their treatment outcomes—are typically limited to the macroscale. Further, given the paucity of human postmortem anatomical data, most microscale features are determined via experimentation in nonhuman primates, most typically monkeys from the genus*Macaca*(macaques). For example, recent evidence suggests that fine-grained differences in structure are critically important for clinical intervention—insights gleaned in monkeys about a human intervention. When deep brain stimulation techniques pioneered byMayberg et al. (2005;Lozano et al., 2008) used for the treatment of intractable depression in humans were replicated in monkeys, a difference of 1 mm from the target area in the subgenual anterior cingulate cortex (Brodmann area 25) completely reversed the effects on whole-brain functional networks (S. H. Fujimoto et al., 2022,^2024^). Effective clinical interventions are, therefore, likely to hinge on efficient translation between cellular and subcellular resolution analyses in animal models and relatively lower resolution*in vivo*evaluations in humans. These two levels of analysis can be bridged by making use of*in vivo*tools in monkey models.

Here, we take the first step in establishing the necessary translation between*in vivo*human imaging studies and postmortem nonhuman animal studies of the insula—a region of rapidly increasing interest across many fields (Craig, 2009;Droutman et al., 2015;Gasquoine, 2014;Singer et al., 2009;Uddin et al., 2017). We employ*in vivo*neuroimaging to evaluate functional and structural connectivity of the macaque insula relative to the established cytoarchitecture and connectivity findings in monkeys (Carmichael & Price, 1994;Evrard, 2019;Evrard et al., 2014;Gallay et al., 2012), what little is known about the cytoarchitecture of the human insula (Bonthius et al., 2005;Kurth, Eickhoff, et al., 2010;Mesulam & Mufson, 1985;Morel et al., 2013;Quabs et al., 2022), and the human imaging literature on the insula (seeCauda et al., 2011;Chang et al., 2013;Deen et al., 2011;Kelly et al., 2012; (Kurth, Zilles, et al., 2010for meta-analyses). The insula is an important target of study because it is characterized by remarkable cytoarchitectural heterogeneity, is now recognized as a critical target for understanding health (seeCraig, 2009;Menon & Uddin, 2010;Uddin et al., 2017for reviews of insula involvement in psychological processes) and disease (e.g., neurodegenerative diseases (Fathy et al., 2020); autism (Uddin & Menon, 2009); anxiety (Chavanne & Robinson, 2021)), and is active in many different psychological processes (Kurth, Zilles, et al., 2010;Menon & Uddin, 2010;Molnar-Szakacs & Uddin, 2022;Uddin et al., 2014,^2017^;Wang et al., 2022).

Accumulating evidence from the human neuroimaging literature widely recognizes functional heterogeneity of the insula and has attempted to map varied functions to different insula subregions. Meta-analyses of human functional magnetic resonance imaging (fMRI) studies suggest the insula can be subdivided into either three or four functional subregions (Cauda et al., 2011;Chang et al., 2013;Kelly et al., 2012;Kurth, Zilles, et al., 2010;Wager & Barrett, 2017) (i.e., boundaries derived from differences in task-based activation or resting-state functional connectivity). In the schema with four regions, the anterior-ventral portion of the insula is implicated in affective and socioemotional processes; the anterior-dorsal portion of the insula is implicated in cognitive processes; a central region (between anterior and posterior) is implicated in chemosensory, olfactory, and gustatory processes; and a posterior region is involved in sensorimotor processes (Kurth, Zilles, et al., 2010) (the three-region schema groups the central chemosensory and anterior-ventral affective regions (Chang et al., 2013)). Analyses of functional and structural connectivity gradients—where differences in connectivity within a given structure are modeled as a continuum of gradual change rather than discrete clusters—in the human insula similarly provide evidence for anterior-posterior and dorsal-ventral axes of organization (Cerliani et al., 2012;Menon et al., 2020;Tian & Zalesky, 2018;Wang et al., 2022) that may be consistent with discrete subregion mapping. The extent to which these observations map to cyto- or myeloarchitectural features of the insula (as would be revealed by postmortem study) is essentially unknown.

Histological studies carried out in macaque monkeys provide critical high-resolution insights into the insula and its cytoarchitecture that are often unavailable from human data where histological analyses are rare and often incomplete (Bonthius et al., 2005;Kurth, Eickhoff, et al., 2010;Morel et al., 2013;Quabs et al., 2022). Macaque histological studies demonstrate that there are three main insula regions that can be differentiated based on the presence and density of granule cells—an anterior-ventral agranular region, a posterior-dorsal granular region, and a transitional dysgranular region found in the mid insula (Carmichael & Price, 1994;Charbonneau et al., 2022;Evrard et al., 2014;Gallay et al., 2012). Within these broad regions, additional subregions can be differentiated based on cytoarchitectural features (Carmichael & Price, 1994;Charbonneau et al., 2022;Evrard et al., 2014;Gallay et al., 2012) and have heterogeneous corticocortical, thalamocortical, and corticosubcortical connectivity (Krockenberger et al., 2023;Mesulam & Mufson, 1982a;Morecraft et al., 2015;Mufson et al., 1981;Mufson & Mesulam, 1982). What little histological evidence exists from a limited number of human cases confirms the patterns observed in macaques and suggests that the presence of small, sharply delimited subregions is a feature of the insula conserved across macaques and humans (Bonthius et al., 2005;Kurth, Eickhoff, et al., 2010;Mesulam & Mufson, 1985;Morel et al., 2013;Quabs et al., 2022). Many open questions remain regarding whether these histological similarities confer translationally relevant similarities that can be measured with*in vivo*neuroimaging.

A clear translational bridge between macaque histological studies and*in vivo*human imaging studies can be constructed by using the same imaging tools available in humans to characterize the macaque insula. Prior*in vivo*imaging studies assessing intrinsic functional networks that contain insula as a hub in the macaque brain suggest some cross-species homologies (Mantini et al., 2011,^2013^;Touroutoglou et al., 2016). For example, we demonstrated that the dorsal and ventral subnetworks of the human “salience network” (Seeley et al., 2007;Touroutoglou et al., 2012) also existed in a small sample (*N*= 4) of awake rhesus monkeys fixating on a screen (Touroutoglou et al., 2016). Whether those findings replicate in a larger sample of monkeys under light anesthesia is not known. Additionally, building a translational bridge between cytoarchitectural studies and human imaging studies requires understanding if intrinsic functional and structural networks of the whole macaque insula mirror the major divisions identified via histological evaluations (Carmichael & Price, 1994;Evrard, 2019;Evrard et al., 2014;Gallay et al., 2012) and in human imaging meta-analyses (Cauda et al., 2011;Chang et al., 2013;Deen et al., 2011;Kelly et al., 2012;Kurth, Zilles, et al., 2010). In the present work, we secure the foundation of this translational bridge via a detailed and multimodal analysis of the macaque insula using the same*in vivo*imaging tools widely available for use with human research subjects and patients.

## 2 Methods

### 2.1 Subjects and living arrangements

Subjects were*N*= 19 adult male rhesus monkeys (*Macaca mulatta*) (age: mean ± SD = 7.6 ± 4.3 years; weight: mean ± SD = 9.3 ± 2.7 kg at the time of MRI acquisition). Monkeys were born and raised in the outdoor field corrals at the California National Primate Research Center (CNPRC) (0.5 acres; 30.5 m × 61.0 m × 2.4 m; approximately 60 to 120 animals per cage). Subjects included all adult male monkeys (greater than 3 years of age) living in one corral who were not actively participating in other investigators’ studies or in the hospital at the time of enrollment for this study. The initial sample consisted of 20 monkeys, however, MRI data were not collected from one animal because of a procedural error. Monkeys were fed monkey chow twice daily, produce at least twice per week, and had*ad lib*access to water. Additional details on rearing and housing can be found in our prior behavioral work with this sample of monkeys (Bliss-Moreau et al., 2021).

All experimental protocols were approved by the University of California Davis Institutional Animal Care and Use Committee and carried out in accordance with the US National Institutes of Health guidelines. All procedures were carried out at the California National Primate Research Center (CNPRC).

### 2.2 MRI data acquisition and processing

Images were acquired on a 3T Siemens Skyra scanner using a custom-built eight-channel head coil optimized for rhesus monkeys (Rapid MR International). Animals were sedated with an initial dose of ketamine (5 mg/kg), intubated, placed in an MR-compatible stereotaxic apparatus, and maintained under 1.0%–1.5% isoflurane. T1-weighted structural volumes were acquired with an MP-RAGE sequence (TR/TE = 2,500/3.65, voxel size = 0.3 × 0.6 × 0.6 mm^3^) followed by acquisition of T2-weighted volumes (TR/TE = 3,000/308, voxel size = 0.4 x 0.8 x 0.8 mm^3^) and a DWI scan using a 2D diffusion-weighted echo planar imaging (EPI) sequence (TR/TE = 8,500/101; voxel size = 0.7 × 0.7 × 1.4 mm^3^; b = 1,200 s/mm^2^). There were 58 noncollinear directions, and 5 nondiffusion weighted images throughout the scan (Jones et al., 1999). The scan was repeated using reverse phase encoding to map geometric distortions induced by the EPI sequence. Monkeys were then injected with the contrast agent ferumoxytol (Feraheme; Amag; 1–7.5 mg/kg) 2 minutes prior to fMRI data acquisition. Two 10-minute long functional scans were acquired (T2*-weighted echo-planar sequence, 260 slices, 84 x 84 in-plane matrix, TR/TE = 2,300/24, flip angle = 80 degrees, voxel size = 1.25 x 1.25 x 1.25 mm^3^). The two functional scans were collected with reversed phase-encode blips, which were used for distortion correction.

#### 2.2.1 T1w and T2w image processing

T1- and T2-weighted images were processed using the CIVET-Macaque pipeline (Lepage et al., 2021), which includes correction for contrast nonuniformities using N3 bias field correction (a critical step for later calculation of T1w/T2w ratio (Glasser et al., 2022)) as well as the generation of a brain mask and cortical surfaces. Manual corrections to cortical surfaces using white matter and cerebrospinal fluid masks were made as needed to ensure the highest quality of surface extraction. Measures of cortical thickness were obtained from the corrected cortical surfaces. To obtain the T1w/T2w ratio mapping, bias-corrected T2w images were first resampled to match the resolution of the T1w images using the*mrtransform*function from the MRtrix software package (Tournier et al., 2019) and then bias-corrected T1w intensity values were divided by bias-corrected T2w intensity values on a voxel-wise basis using MRtrx’s*mrcalc*function.

#### 2.2.2 fMRI processing

For seed-based functional connectivity analyses, images were preprocessed using procedures identical to those previously published (Mantini et al., 2011) (such that preprocessing methods would mirror those inTouroutoglou et al. (2016), which we sought to replicate with our first seed-based analysis assessing differences in functional connectivity between the dorsal and ventral anterior insula shown inFig. 1). BOLD images were collected with reversed phase-encode blips, resulting in pairs of images with distortions going in opposite directions. From these pairs of images, the susceptibility-induced off-resonance field was estimated using the “topup” procedure in FSL (Jenkinson et al., 2012), resulting in a single corrected image (Andersson et al., 2003;Smith et al., 2004). After the BOLD images were corrected for distortions in FSL, we performed the following steps using SPM12.0 (Friston et al., 2007): (1) slice-timing correction, (2) motion correction, (3) linear detrending of BOLD signal, (4) regressing cerebrospinal, white matter signals, and their temporal derivatives out of the BOLD signal (5) coregistration of functional images to the subjects’ native anatomical image, (6) spatial normalization to macaque F99 atlas space using the 112RM template (McLaren et al., 2009), and (7) spatial smoothing with a Gaussian kernel at 2 mm full-width-half-maximum (FWHM). As inTouroutoglou et al. (2016), we took a more conservative approach and did not perform global signal regression, which potentially induces spurious negative correlations in intrinsic correlation between regions (Murphy & Fox, 2017).

**Fig. 1.**
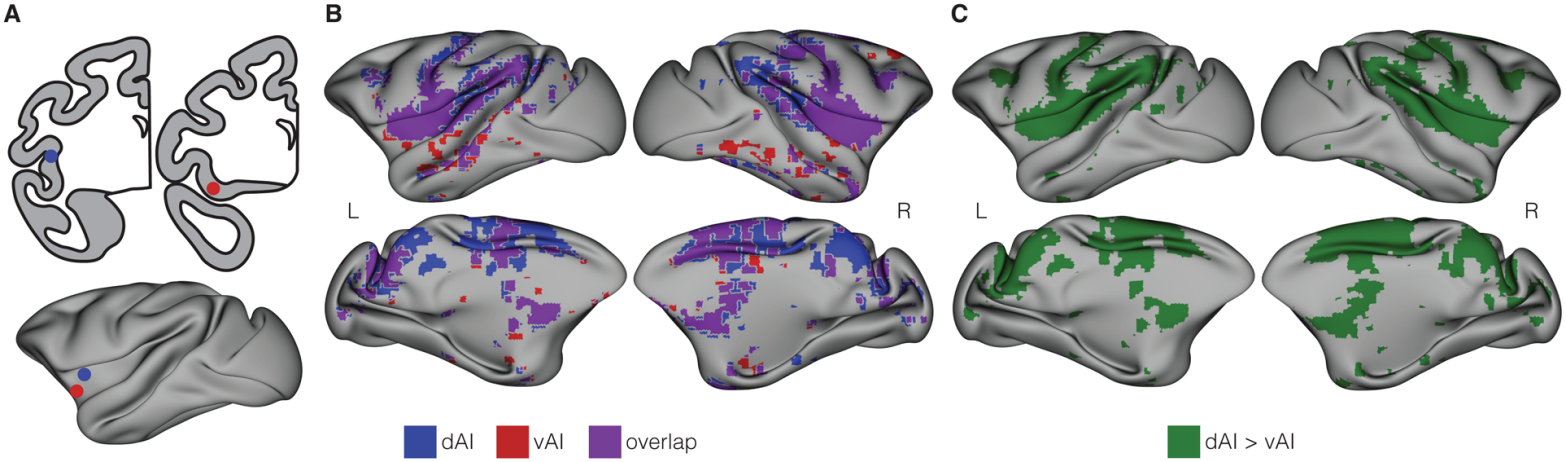
Functional connectivity of the dorsal and ventral anterior insula. (A) Location of 2 mm spherical seeds for intrinsic functional connectivity analysis. Seed placement is demonstrated on schematics of coronal sections (top) and on an inflated cortical surface (bottom) shown from a lateral view. The ventral anterior insula seed (red) was placed at x = 17.5, y = 5.5, z = -3.5. The dorsal anterior insula seed (blue) was placed at x = 20, y = 2.5, z = 1.5. Coordinates are derived from the F99 atlas (McLaren et al., 2009). Placement is identical to the seeds from our prior report (Touroutoglou et al., 2016). (B) 3D surface projection of intrinsic functional connectivity maps. Maps of the dorsal anterior insula (dAI: blue) and ventral anterior insula (vAI: red) functional connectivity were projected onto inflated cortical surfaces, each shown at a voxel-wise intensity threshold of*p*≤ .001, corrected for false discovery rate. Note that the vAI network is almost entirely contained within the dorsal anterior insula network (areas of overlap are shown in purple). (C) Cortical regions where the dAI seed exhibited significantly stronger connectivity compared with the vAI seed. Within each subject, the difference between connectivity with the dAI seed and the vAI seed was computed at each voxel [r(dAI) - r(vAI)] and these difference maps underwent permutation testing using FSL’s randomize function. Statistical maps were thresholded at*p*< .05 voxel-wise, with family-wise error corrected for multiple comparisons. No cortical region exhibited significantly stronger intrinsic functional connectivity with the vAI seed than the dAI seed.

For gradient-based analyses, images were preprocessed using a custom version of the AFNI NHP processing pipeline (A. Fujimoto et al., 2022;Jung et al., 2021) to align with other ongoing work in our laboratory. Images were slice time corrected, motion corrected, aligned with the T1-weighted image, and warped to the standard space. Distortion correction was accomplished using reverse phase encoding (i.e., reverse blip). Following alignment to standard space, EPIs were blurred using a 2 mm FWHM filter and rescaled to reflect percentage signal change from baseline. We then regressed the motion derivatives from each scan along with cerebrospinal fluid and white matter signal regressors.

Following statistical analyses (discussed below), group statistic images and gradients were projected onto the surface space using connectome workbench’s*volume-to-surface mapping*(with trilinear interpolation) for visualization (Marcus et al., 2011). For analyses replicatingTouroutoglou et al. (2016; i.e.,Fig. 1), results are shown on F99 (Van Essen et al., 2012) surfaces to take advantage of standard coordinate mappings and ensure comparability across studies. All other surface-based visualizations are shown on NMT (Jung et al., 2021) surfaces.

#### 2.2.3 dMRI processing

Diffusion image preprocessing was accomplished using the open-source software package MRtrix3 (Tournier et al., 2019). We adapted our previous pipeline (Raven et al., 2023) to include the following steps: image denoising (Veraart et al., 2016), Gibbs ringing (Kellner et al., 2016), gradient checking (Jeurissen et al., 2014), correction of movement artifacts, and geometric susceptibility and eddy current distortions (Andersson & Sotiropoulos, 2016). Using the preprocessed images, diffusion tensors were fit to each voxel, and parametric maps were computed for fractional anisotropy (FA) and mean diffusivity (MD) (Le Bihan et al., 2001). FA is calculated from the eigenvalues of the diffusion tensor, and representative of fiber architecture, including packing of cellular structures and their distribution and orientation within tissue (Jones et al., 2013). MD is the mean of the three eigenvalues of the diffusion tensor, and relates to the size, density, and structure of space within tissue.

FA and MD maps were aligned and resampled to the subjects’ native anatomical image using ITK-SNAP (Yushkevich et al., 2019). To mitigate partial volumes at the boundary of the pial surface, surfaces were resampled to remove the outer 30% of the cortical ribbon (Whitaker et al., 2016). Parametric maps were then projected onto cortical surfaces using Connectome Workbench’s*volume-to-surface mapping*(Marcus et al., 2011).

### 2.3 Seed-based functional connectivity analyses

**Fig. 2.**
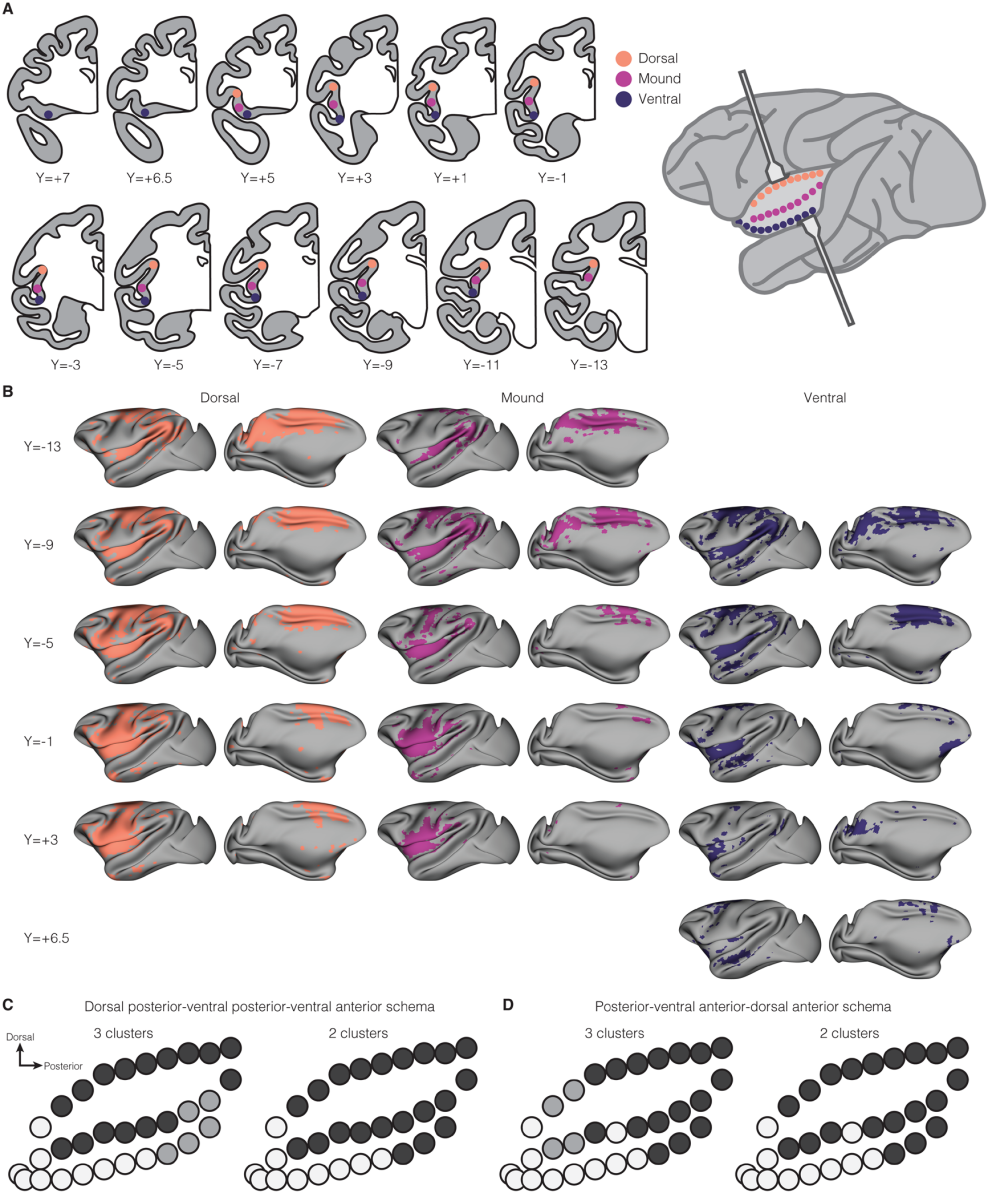
Clustering of functional connectivity throughout the macaque insula. (A) Coronal sections show the placement of 31 seeds tiling the dorsal fundus (orange), mound (magenta), and ventral fundus (blue) of the insula. Each seed was a 2 mm diameter sphere placed within the cortical ribbon. A schematic representation of the macaque brain with the frontoparietal and temporal opercula pulled back to reveal the insula shows approximate placement of the 31 seeds along the surface of the insula. (B) Statistical maps showing the topography and magnitude of intrinsic functional connectivity of every other insula seed from (A) with the rest of the brain on an inflated surface of the macaque brain. Left hemispheres are shown from a lateral and medial view as seeds were placed in the left hemisphere. The pattern of functional connectivity in the right hemisphere was comparable with that in the left hemisphere in all cases. The coordinate to the left of each row corresponds to the coordinates shown on the coronal sections in (A). Connectivity maps of the dorsal seeds are shown in orange (left), mound seeds in magenta (middle), and ventral seeds in blue (right). (C) A representative example (variable entropy similarity measure) of the “dorsal posterior-ventral posterior-ventral anterior” clustering solution portrayed on a 2-dimensional schematic of the 31 insula seeds. On the left, the three-cluster solution is shown with each seed colored according to its cluster membership (dark gray, gray, light gray). On the right, the two-cluster solution is shown with each seed colored according to its cluster membership (dark gray, light gray). (D) A representative example (inverse occurrence frequency similarity measure) of the “posterior-ventral anterior-dorsal anterior” clustering solution, portrayed as described in (C).

Functional connectivity analyses were conducted using a seed-based approach with 2 mm spherical seeds. In our first analysis focused on the anterior insula, seeds were placed at the same coordinates as those inTouroutoglou et al. (2016)for the dorsal anterior insula (dAI: 20, 2.5, 1.5) and ventral anterior insula (vAI: 17.5, 5.5, -3.5) (Fig. 1A). In our second analysis including the whole insula, seeds were tiled along the dorsal-ventral and anterior-posterior extents of the insula in the left hemisphere (Fig. 2A). The spatial topography of each seed’s functional connectivity was determined using whole-brain seed-to-voxel correlations of BOLD timeseries (Mantini et al., 2011;Touroutoglou et al., 2016). The Pearson correlation between the BOLD timeseries in each voxel and the average BOLD timeseries in each insula seed served as the measure of functional connectivity. Differing fromTouroutoglou et al. (2016), subject-level effective degrees of freedom for intrinsic correlations were adjusted for autocorrelation using the method developed byAfyouni et al. (2019). Group connectivity maps were computed using a random-effects analysis and only positive correlations were analyzed as the dorsal and ventral salience networks as well as interoceptive-allostatic network have been shown to have positive intrinsic correlations (Kleckner et al., 2017;Touroutoglou et al., 2012). Significance was set to a voxel-wise threshold of T > 3.61, corresponding to a false-discovery rate (FDR) of*q*≤ 0.001.

In order to facilitate describing the voxel-wise results, coordinates of peak functional connectivity were identified using FSL’s*cluster*tool, and anatomical areas to which these coordinates corresponded were manually identified in ITK-SNAP 3.6.0 (Yushkevich et al., 2019) on the 112RM-SL reference image registered to an anatomical atlas (Paxinos et al., 2000;Saleem & Logothetis, 2012). The exact coordinates and locations are provided inSupplementary Tables S1 and S2.

#### 2.3.1 Differences in voxel-wise connectivity strength between vAI and dAI networks

In order to test for differences in functional connectivity strength between the ventral and dorsal anterior insula seeds throughout the brain, within each subject, we computed voxel-wise difference maps of vAI correlation strength subtracted from dAI correlation strength [r(dAI)— r(vAI)]. Next, we used FSL’s randomize function (with 10,000 permutations) (Smith et al., 2004) to compute maps identifying voxels which exhibited significantly stronger correlation with the dAI seed and the vAI seed, respectively. These maps underwent threshold free cluster enhancement (TFCE), were thresholded at a 5% level of significance adjusting for multiple comparisons, and were then thresholded to include only voxels which exhibited significant correlations in the respective vAI and dAI maps.

### 2.4 Hierarchical nominal clustering analyses

Hierarchical clustering analyses were carried out in R version 4.3.1 (R Core Team, 2023) using the*nomclust*package (Šulc et al., 2022). For each seed, the peak of each activation cluster was identified using a rhesus macaque atlas (Saleem & Logothetis, 2012). A matrix was then constructed in which each column represented an insula seed and each row represented a region with which a seed might be connected (i.e., all of the target regions identified across all 31 seeds). Each cell of this matrix contained either a 0 or 1, indicating whether a given seed was functionally connected to a given target region. Target regions were collapsed across hemisphere (such that connectivity with, for example, the left amygdala was considered the same as connectivity with the right amygdala or the amygdala in both hemispheres). In this way, each seed’s column in the matrix represented its connectivity profile with the rest of the brain. As labeled target regions were categorical variables, methods for hierarchical clustering analysis of nominal variables were used to analyze the clustering of insula seeds according to their connectivity. Twelve different similarity measures (Šulc et al., 2022) (i.e., functions used to compute the proximity matrix) were used to cluster the 31 seeds. Across each of these measures, six evaluation criteria were used to determine the optimal number of clusters (pseudo F index based on the mutability [PSFM], pseudo F index based on the entropy [PSFE], Bayesian information criteria [BIC], Akaike information criteria [AIC], best*k*[BK] index, and silhouette index [SI]), allowing for evaluation based on variability (PSFE, PSFM, BK), likelihood (BIC, AIC), and distance (SI). The average linkage method was used for all analyses, which considers the pair-wise dissimilarity between objects in different clusters to avoid both the chaining phenomenon (wherein clusters are merged based on the closest elements but other elements are very distant) and sensitivity to outliers (Šulc & Řezanková, 2019).

### 2.5 Gradient analyses with diffusion map embedding

Gradients of extrainsular functional connectivity, intrainsular functional connectivity, cortical thickness, T1w/T2w ratio, fractional anisotropy, and mean diffusivity were derived using diffusion map embedding (Coifman & Lafon, 2006;Vos De Wael et al., 2020). Diffusion map embedding is a technique for nonlinear data dimensionality reduction. This method enables an assessment of the organization of the similarity between large number of data points by identifying a set of low-dimensional manifolds that capture principal dimensions of the spatial variation in each metric. For extrainsular and intrainsular functional connectivity, initial computation of the affinity matrix was done in volume space according to cosine similarity and then used as input to diffusion map embedding. The resulting volumes were then projected onto surface space using connectome workbench’s*volume-to-surface mapping*as previously described. For FA, MD, thickness, and T1w/T2w ratio, metrics were already in surface space before computation of the affinity matrix was carried out based on cosine similarity. These affinity matrices were then also used as input to diffusion map embedding. Diffusion map embedding yielded 10 gradients per affinity matrix. Here, we focus on the two dominant gradients as they explained together more than 50% of the variance for each metric, and no gradient beyond the two dominant gradients individually captured more than 15% of the variance (seeSupplementary Fig. S1for scree plots).

## 3 Results

### 3.1 An intrinsic functional network anchored in the macaque anterior insula

The insula is a hub in multiple intrinsic (or resting-state) functional networks identified in human (Menon & Uddin, 2010;Molnar-Szakacs & Uddin, 2022) and macaque (Mantini et al., 2011,^2013^;Touroutoglou et al., 2016;Vincent et al., 2008) neuroimaging data. Perhaps most importantly, it plays a role in processing salient affective information and regulates interoception and allostasis—processes critical for survival and consequential for affective/emotional health and a variety of disease states (Uddin, 2015). Early conceptions of the network regulating salience detection and allostatic processing focused on activity in the anterior portion of the insula and studies in humans demonstrated dissociable networks anchored in the dorsal dysgranular and ventral agranular anterior insula (Seeley et al., 2007;Touroutoglou et al., 2012). From macaque-tracing studies, the dorsal anterior insula is known to be reciprocally connected with prefrontal, parietal, and temporal regions while ventral anterior insula is connected primarily with anterior cingulate cortex, entorhinal cortex, and lateral orbitofrontal cortex (Mesulam & Mufson, 1982a,1982b,^1985^). We previously identified similar networks in rhesus monkeys (Touroutoglou et al., 2016), although the network we identified originating at the macaque dorsal anterior insula did not have strong functional connectivity with frontal and parietal regions characteristic of this region in humans (Touroutoglou et al., 2012). However, this cross-species dissimilarity may be explained by the fact that monkeys in our sample were in a different state (under light isoflurane anesthesia compared with awake and attending to a stimulus in the prior study). Further, the sample in our prior work (Touroutoglou et al., 2016) included only four monkeys and so the extent to which the observed patterns of functional connectivity that constitute the networks anchored in the anterior insula hold in a larger sample is not known. To evaluate this, we performed the same seed-based analysis of functional connectivity as in our prior study with a sample of*N*= 19 group reared and housed adult male rhesus monkeys (aged 3–14 years, mean ± SD = 8.1 ± 4.7) living in one of the large field enclosures at the California National Primate Research Center while they were lightly sedated with isoflurane (~1%). We placed identically positioned seeds (Touroutoglou et al., 2016) in the ventral (F99 coordinates: x = 17.5, y = 5.5, z = -3.5) and dorsal (F99 coordinates: x = 20, y = 2.5, z = 1.5) anterior insula (seeFig. 1) to evaluate their intrinsic functional connectivity with the rest of the brain.

#### 3.1.1 Intrinsic functional connectivity within the ventral anterior insula

The ventral anterior insula (vAI) network is shown inFigure 1Aand the regions exhibiting significant connectivity with the vAI are detailed inSupplementary Table S1. This analysis largely replicated the network identified in our prior report (Touroutoglou et al., 2016). Present in this network were regions traditionally included in the human ventral salience network, including the anterior and mid cingulate cortex (dorsal ACC (dACC), subgenual ACC (sgACC) and mid cingulate cortex (MCC)), orbitofrontal cortex (OFC), amygdala, striatum, and bilateral anterior insula. We also replicated previously observed differences between the human and rhesus ventral salience networks (Touroutoglou et al., 2016). The rhesus ventral salience network included portions of prefrontal cortex (arcuate sulcus and caudal principal sulcus (areas 44, 45, and 46v)), frontal cortex (e.g., central sulcus), mid insula, superior temporal sulcus, and striate cortex—regions not typically included in the human ventral network. The precuneus, bilateral intraparietal sulcus, and temporoparietal areas also showed significant connectivity with the vAI—departures from our prior report (Touroutoglou et al., 2016).

#### 3.1.2 Intrinsic functional connectivity within the dorsal anterior insula

The dorsal anterior insula (dAI) network is shown inFigure 1Band the regions exhibiting significant connectivity with the dAI are detailed inSupplementary Table S2. These findings partially replicate our prior report (Touroutoglou et al., 2016), including the identification of strong connectivity between dAI and the posterior cingulate cortex (PCC), sgACC, dACC, and MCC (differing from cingulate targets in the human dorsal network, which traditionally include only dACC and MCC). Further, we again identified extensive bilateral intrinsic connectivity with the putamen, which is absent in the human dorsal salience network. In contrast with our prior report, our current analyses show extensive bilateral connectivity between dAI and the intraparietal sulcus, inferior parietal lobule, and frontal targets (including the caudal principal sulcus and ventral arcuate sulcus)—aligning the present results more closely with the human dorsal salience network (Seeley et al., 2007;Touroutoglou et al., 2012) than our prior findings (Touroutoglou et al., 2016).

#### 3.1.3 No evidence of statistically dissociable anterior insula subnetworks in macaques

While our vAI and dAI networks largely recapitulated the networks we previously identified in an independent sample of monkeys (Touroutoglou et al., 2016), their topographies were essentially overlapping in this sample: the vAI network emerged as a subset of larger regions constituting the dAI network. This finding suggested that the two networks were not dissociable in our sample, in contrast with our prior finding that the vAI and dAI networks were statistically dissociable (i.e., within-network correlations were significantly stronger than between-network correlations) (Touroutoglou et al., 2016). To investigate this empirically, we computed the connectivity strength difference map [r(dAI)— r(vAI)] shown inFigure 1C. After adjusting for multiple comparisons, there were no voxels found to exhibit stronger correlations with the vAI compared with dAI seed, other than those in the immediate vicinity of the seed (likely because of spatial autocorrelation), failing to provide evidence for statistical dissociability of these networks.

### 3.2 Intrinsic functional connectivity throughout the macaque insula

Tract-tracing studies in monkeys (Augustine, 1996;Friedman et al., 1986;Mesulam & Mufson, 1982b,^1985^) and electrical stimulation and recordings in human patients (Almashaikhi et al., 2014) demonstrate that inputs to the insula first arrive in the posterior and mid dorsal portions of the structure before undergoing integration and refinement via intrainsular circuitry, ultimately arriving in the anterior portion. To better understand functions of the anterior insula, it follows that it is necessary to understand the network involvement of the rest of the structure and intrainsular circuitry. To further characterize homologies between human and macaque insular networks, we expanded our seed-based approach to tile the extent of the insula with 31 2-mm spherical seeds, organized along the dorsal-ventral and anterior-posterior axes (seeFig. 2A;Supplementary Fig. S2). We placed 10 seeds along the dorsal fundus of the insula (i.e., the superior limiting sulcus; orange inFig. 2A), 10 along the middle of the insula (i.e., the “mound” region; magenta inFig. 2A), and 11 along the ventral fundus and opercular region of the insula (i.e., the inferior limiting sulcus and ventral surface; blue inFig. 2A). Peaks of functional connectivity maps with the rest of the brain associated with each of the 31 seeds were labeled by identifying the peak in a standard rhesus macaque atlas (Saleem & Logothetis, 2012) and then data were analyzed using hierarchical cluster analysis (*nomclust*(Šulc et al., 2022;Sulc & Cibulkova, 2021) package in R version 4.0.2 (R Core Team, 2023)). Connectivity of select seeds is shown on inflated cortical surfaces inFigure 2B(seeSupplementary Fig. S2for all 31 seeds).

#### 3.2.1 Hierarchical clustering of insular functional connectivity

We assessed clustering of insula subregions based on whole-brain intrinsic functional connectivity data using 12 different similarity measures (Šulc et al., 2022) (i.e., functions used to compute the proximity matrix) and according to 6 evaluation criteria (pseudo F index based on the mutability [PSFM], pseudo F index based on the entropy [PSFE], Bayesian information criteria [BIC], Akaike information criteria [AIC], best*k*[BK] index, and silhouette index [SI]) allowing for evaluation based on variability (PSFE, PSFM, BK), likelihood (BIC, AIC), and distance (SI). The optimal number of clusters determined according to similarity measure and evaluation criteria is presented inTable 1. Across similarity measures, evaluation criteria largely suggested that between 2 and 5 clusters fit the data best, with exception of BIC which always suggested 1 cluster. Clustering solutions with two and three clusters were determined to be optimal most frequently and so we provide a visualization of representative two and three cluster solutions shown inFigure 2C and D(seeSupplementary Fig. S3for all clustering solutions). There was some expected heterogeneity in cluster identity for individual seeds across these solutions. This variability is consistent with the human literature where variation in the clustering of insula subregions is highly variable and seems to be related to methodological features such as model parameters (Nanetti et al., 2009;Uddin et al., 2014). This heterogeneity also likely reflects our use of several different similarity measures and evaluation criteria.

**Table 1.** Optimal number of clusters suggested by hierarchical clustering.

Similarity measure	PSFM	PSFE	BIC	AIC	BK	SI
Eskin	**3**	4	1	**3**	4	6
Goodall 1	**2**	**2**	1	**2**	**2**	5
Goodall 2	**2**	**2**	1	4	4	**2**
Goodall 3	**3**	**3**	1	**3**	5	5
Goodall 4	5	5	1	1	5	**2**
Inverse occurrence frequency	**2**	**2**	1	**2**	**2**	**2**
Occurrence frequency	**2**	**2**	1	**3**	4	6
Lin	**2**	**2**	1	**3**	5	**2**
Lin 1	**2**	**2**	1	**3**	5	**2**
Simple matching coefficient	**2**	**3**	1	**3**	**3**	**2**
Variable entropy	**2**	**2**	1	**3**	**3**	**2**
Variable mutability	**2**	**2**	1	**3**	**3**	**2**

Note: The optimal number of clusters was assessed using 6 evaluation criteria (pseudo F index based on the mutability [PSFM], pseudo F index based on the entropy [PSFE], Bayesian information criteria [BIC], Akaike information criteria [AIC], best*k*[BK] index, and silhouette index [SI]). This allowed for evaluation based on multiple features of the data including variability (as in PSFE, PSFM, and BK), likelihood (as in BIC, AIC), and distance (as in SI). As 2 and 3 cluster solutions (bold) were most often found to be optimal, we present two representative clustering solutions inFigs. 2C-D.

Across these different clustering solutions, there were two overarching organization schemas, described in detail below. The first schema included posterior, ventral anterior, and dorsal anterior clusters (similarity measures: Eskin, Goodall 1, inverse occurrence frequency;Fig. 2C). The second schema included dorsal posterior, ventral posterior, and ventral anterior clusters (Goodall 3, occurrence frequency, Lin, Lin 1, simple matching coefficient, variable entropy, variable mutability;Fig. 2D). Two-cluster solutions almost invariably (with the exception of Eskin) divided the structure into dorsal posterior and ventral anterior subregions. This was driven by the fact that ventral anterior seeds most often clustered together and dorsal posterior seeds most often clustered together, consistent with prior findings in humans which found, using repeated*k*-means clustering, that a large zone in the middle insula could not be reliably classified (Nanetti et al., 2009).

As has been observed in studies assessing the “functional fingerprints” of human insula subregions via fMRI (Uddin et al., 2014), there was considerable overlap between clusters in terms of their connectivity. Most, or all, seeds were connected to sensory cortex (somatosensory, auditory, visual), motor cortex (primary, premotor), dorsal striatum (caudate, putamen), superior temporal cortex, orbitofrontal cortex (areas 11, 12, 13), anterior cingulate cortex, amygdala, hypothalamus, and agranular insula—highlighting the role of the entire insula in sensorimotor integration and visceromotor/autonomic processing, planning, and control. While there was overlap in connectivity, each subregion did have some distinct connectivity patterns, which we describe below.

In the first three-cluster schema (C1; dorsal posterior, ventral posterior, and ventral anterior;Fig. 2C), the seeds belonging to the dorsal posterior cluster, but not the ventral posterior or ventral anterior clusters, were connected to the thalamus. This connectivity is consistent with the role of the insula’s dorsal fundus as the primary interoceptive cortex defined by its granular cytoarchitecture and thalamic inputs (Evrard, 2019;Evrard et al., 2014). Additionally, there was no evidence of connectivity between the dorsal posterior cluster and frontal cortical regions (areas 10, 14, and 44), temporal regions (i.e., perirhinal, entorhinal cortex), and presupplementary motor cortex—regions with which the ventral posterior and/or ventral anterior clusters were connected.

The ventral posterior and ventral anterior clusters in C1 were both connected to frontal regions (areas 10, 14, and 44). Information in the ventral portion of the insula is thought to have undergone significant processing via the cascade that begins at the dorsal posterior extent (top-back) and moves forward to the ventral anterior extent (bottom-front), which we refer to as the forward-downward insular cascade. As such, the connection between the ventral clusters and frontal cortex regions supports the idea that that insulaprefrontal connections communicate primarily information that has already undergone significant intrainsular processing.

Connectivity with dorsolateral prefrontal cortex (area 46)—a canonical “cognitive” area of the brain—was limited to the dorsal posterior and ventral posterior clusters and was not present in the ventral anterior. Further, the ventral anterior cluster was the only cluster of the three which exhibited connectivity with the anterior olfactory nucleus and olfactory tubercles, potentially implicating this cluster in specific processing of olfactory information. There were a greater number of seeds in the ventral anterior cluster (relative to the posterior clusters) which were connected to the ventral striatum, including the nucleus accumbens. The two ventral clusters were connected to the perirhinal and entorhinal cortex which may also imply some functional specificity in visual and/or memory processing in this portion of the insula. That said, in this schema, there was no clear clustering of connectivity with the hippocampus and nearby amygdala–hippocampus region. The two-cluster solution for C1 collapsed the ventral posterior and dorsal posterior clusters together to form a ventral anterior-dorsal posterior division, consistent with the bipartition of insula function previously suggested for the human insula (Cauda et al., 2011).

The second three-cluster solution (C2) included a posterior, a dorsal anterior, and a ventral anterior cluster (Fig. 2D). The dorsal anterior cluster, but not the other clusters, was characterized by connectivity with the hippocampus, potentially related to this subregion’s canonical association with cognition from the human fMRI literature (Kurth, Zilles, et al., 2010). This dorsal anterior subregion was also characterized by lack of connectivity with claustrum, cerebellum, and portions of the temporal cortex (area TF, area TFO, medial superior temporal area, middle temporal area). The ventral anterior and posterior subregion patterns of connectivity were otherwise similar to the ventral anterior and dorsal posterior subregions, respectively, in C1. The two-cluster solution corresponding to C2 collapsed together the dorsal anterior and dorsal posterior clusters to form a ventral anterior-dorsal posterior division as was true with C1.

### 3.3 Structural connectivity of the macaque insula

**Fig. 3.**
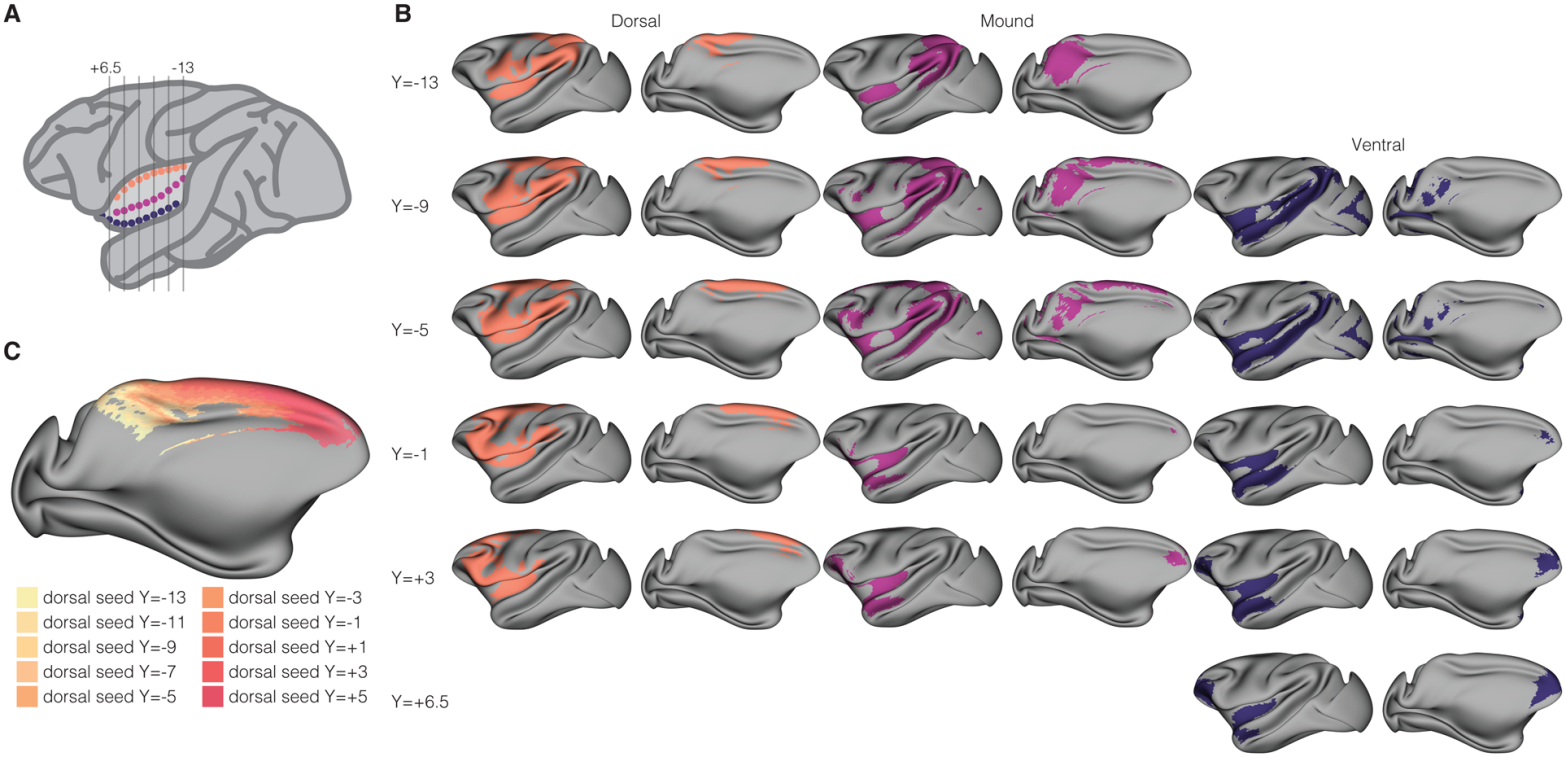
Structural connectivity throughout the macaque insula. (A) Schematic of seed positioning as shown inFigure 2A. (B) Maps showing the end points of seed-based tractography analyses projected onto cortical surfaces. Left hemispheres are shown from lateral and medial views for every other seed. The coordinate to the left of each row corresponds to the seed location in (A). Identical seed locations were used as shown inFigure 2Band maps are shown for the same seeds allowing for comparison across the functional and structural domains for each seed. Maps for dorsal seeds are shown in orange (left), mound in magenta (middle), and ventral in blue (right). (C) Topographic connectivity between the dorsal fundus of the insula and the overlying cingulate and motor cortex. End points of seed-based tractography analyses are shown on a medial view of the cortical surface. End point projections for all 10 dorsal seeds are shown ranging from light yellow (most posterior dorsal insula seed) to pink (most anterior dorsal insula seed).

To complement our seed-based analysis of the intrinsic functional connectivity of the macaque insula, we carried out a similar analysis, using 31 identically placed seeds (Fig. 3A) to assess the structural connectivity of the macaque insula as revealed by diffusion MRI (dMRI). This analysis serves as a bridge between invasive track-tracing studies which can only be carried out in macaques (and not humans; e.g.,Mesulam & Mufson, 1982a,1982b;Mufson & Mesulam, 1982;Pandya et al., 1981;Vogt et al., 1987;Vogt & Pandya, 1987) and diffusion imaging studies carried out in humans (e.g.,Cerliani et al., 2012;Cloutman et al., 2012;Jakab et al., 2012), which are used to infer information about white matter tracks in the absence of tracer data. We found evidence of broad connectivity of macaque insula with the rest of the brain, including regions in the frontal, temporal, parietal, and occipital lobes—consistent with both prior macaque tract-tracing and human diffusion findings (Fig. 3B;Supplementary Fig. S4). Supporting our intrinsic functional connectivity analyses (described above), we also found evidence that some areas of insula are uniquely connected to some other neural regions.

#### 3.3.1 Structural connectivity along the dorsal fundus of the insula

Ten of the 31 seeds tiled the dorsal fundus of the insula, revealing a profile primarily consisting of connectivity with the overlying parietal and frontal opercula (shown in orange inFig. 3A, B;Supplementary Fig. S4). All dorsal seeds were connected to somatosensory, motor, and other insular regions. Connectivity with somatosensory regions included primary, secondary, and higher order somatosensory cortex (i.e., somatosensory areas 3a and 3b) and motor regions included primary motor cortex, dorsal and ventral premotor cortex, and supplementary motor area. Along the midline, the entire dorsal fundus was connected to the entire cingulate cortex in a topographic manner such that more posterior seeds were connected with posterior and mid cingulate, while more anterior seeds were connected with anterior cingulate cortex (Fig. 3C). The seeds located at the posterior extent of the insula were selectively connected (i.e., more anterior seeds did not have this connectivity) to extensive swaths of parietal cortex, consistent with the multisensory and vestibular functionality attributed to this region across humans and monkeys (Evrard, 2019). The seeds in the anterior dorsal extent, in contrast, had connections with lateral prefrontal cortex that were not seen in the more posterior dorsal seeds, consistent with the more “cognitive” characterization of the function of this region (Menon & Uddin, 2010;Uddin et al., 2017).

#### 3.3.2 Structural connectivity along the mound of the insula

Ten seeds tiled the “mound” region of the macaque insula—which has been described as an “incipient ventral gyrus” (Evrard et al., 2014)—between the dorsal and ventral extents of the insula (shown in magenta inFig. 3A, B;Supplementary Fig. S4). Like the dorsal seeds, mound seeds were also connected to the whole insula. Extrainsular connectivity differed from the dorsal seeds, however, as connectivity with somatosensory and motor regions was much sparser when present. Mound seed connectivity was more heterogeneous than dorsal seed connectivity, as posterior seeds were connected primarily with parietal cortex, including extensive connectivity with medial parietal regions (e.g., area 7 m) that was not apparent in any of the posterior dorsal seeds. More anterior mound seeds had no connectivity with parietal cortex but rather were connected to premotor and lateral prefrontal regions. Mound seeds were also connected to temporal regions. More posterior seeds were connected to most of the superior temporal sulcus and more mound seeds at the anterior extent were connected to more anterior temporal regions that were largely nonoverlapping with the STS connectivity of the more posterior seeds. As with the dorsal seeds, there mound seeds were connected to cingulate cortex throughout, again in a topographic manner. The farthest anterior mound seeds had more elaborate connections with the anterior cingulate cortex than the farthest anterior dorsal seeds.

#### 3.3.3 Structural connectivity along the ventral fundus of the insula

The final 11 seeds tiled the ventral fundus of the macaque insula, extending onto the orbital surface where agranular anterior insula meets posterior orbitofrontal cortex (shown in blue inFig. 3A, B;Supplementary Fig. S4). As with the dorsal and mound seeds, ventral seeds were connected to the entire insula. The connectivity of the posterior ventral seeds was similar to that of the posterior mound seeds, including connectivity with parietal regions and superior temporal sulcus. Ventral seeds lacked connectivity with somatosensory and motor regions. However, more posterior ventral seeds were connected with visual regions, including V2, perhaps including fiber tracts from large bundles running along the anterior-posterior axis of the brain (as insula is not known to exhibit such connectivity in tract-tracing data). More anterior ventral seeds were characterized by connectivity with the superior temporal gyrus as well as unique connectivity with the frontal pole. There was also extensive connectivity between ventral anterior seeds and anterior cingulate cortex extending down into the subgenual cingulate region—which differed from the connectivity of the mound seeds.

### 3.4 Gradients of insular organization

Our seed-based analyses of intrinsic functional and structural connectivity data in the macaque insula suggested that this structure may display patterns of connectivity similar to the human insula. However, we observed variance in the clustering of seed-based connectivity. Although future investigations in macaques, particularly with sample sizes approaching those similar to the Human Connectome Project, may allow for more conclusive clustering, one possibility is that the parcellation of insula into discrete functional subregions is not the most useful approach to understanding its organization—as has been suggested previously in the human insula literature (Cerliani et al., 2012;Nanetti et al., 2009;Uddin et al., 2014). To reduce the high dimensionality of our data, we characterized the dominant organizing gradients of insula functional connectivity and other structural features accessible by*in vivo*imaging (cortical thickness, T1w/T2w ratio, fractional anisotropy, and mean diffusivity) as has been done previously (Katsumi et al., 2023;Royer et al., 2020;Vos De Wael et al., 2020). This allows for the assessment of whether different measures provide similar information about organizational principles and also whether*in vivo*metrics in macaques reveal similar axes of organization to the macaque histological literature and human imaging literature. Histology data from monkeys indicate a posterior dorsal to anterior ventral shift in cytoarchitecture from granular to dysgranular to agranular cortex (Charbonneau et al., 2022;Evrard et al., 2014;Gallay et al., 2012;Mesulam & Mufson, 1982a) (supporting the forward-downward information processing cascade), as well as anterior-posterior gradients in cortical myelin (Mesulam & Mufson, 1982a) and dorsal-ventral gradients in other cellular properties including myelin content and density of parvalbumin and acetylcholinesterase containing fibers (Gallay et al., 2012). Similarly, fMRI and dMRI data from humans show gradients of functional and structural connectivity as well as myelin content along the anterior-posterior and dorsal-ventral axes (Menon et al., 2020;Royer et al., 2020;Veréb et al., 2021;Wang et al., 2022). We used diffusion map embedding—an established technique for nonlinear dimensionality reduction which has frequently been applied to neuroimaging data (Katsumi et al., 2023;Vos De Wael et al., 2020)—to characterize gradients of intrainsular functional connectivity (i.e., between a given pair of voxels within the insula), extrainsular functional connectivity (i.e., between the insula and the rest of the brain), fractional anisotropy (FA; dMRI anisotropy of water molecules within voxel), mean diffusivity (MD; dMRI total diffusion within voxel), cortical thickness (thickness of the cortical gray matter), and T1w/T2w ratio (the ratio of the T1w and T2w images, a proxy for myelin content (Glasser & Van Essen, 2011)).

#### 3.4.1 Functional connectivity gradients

**Fig. 4.**
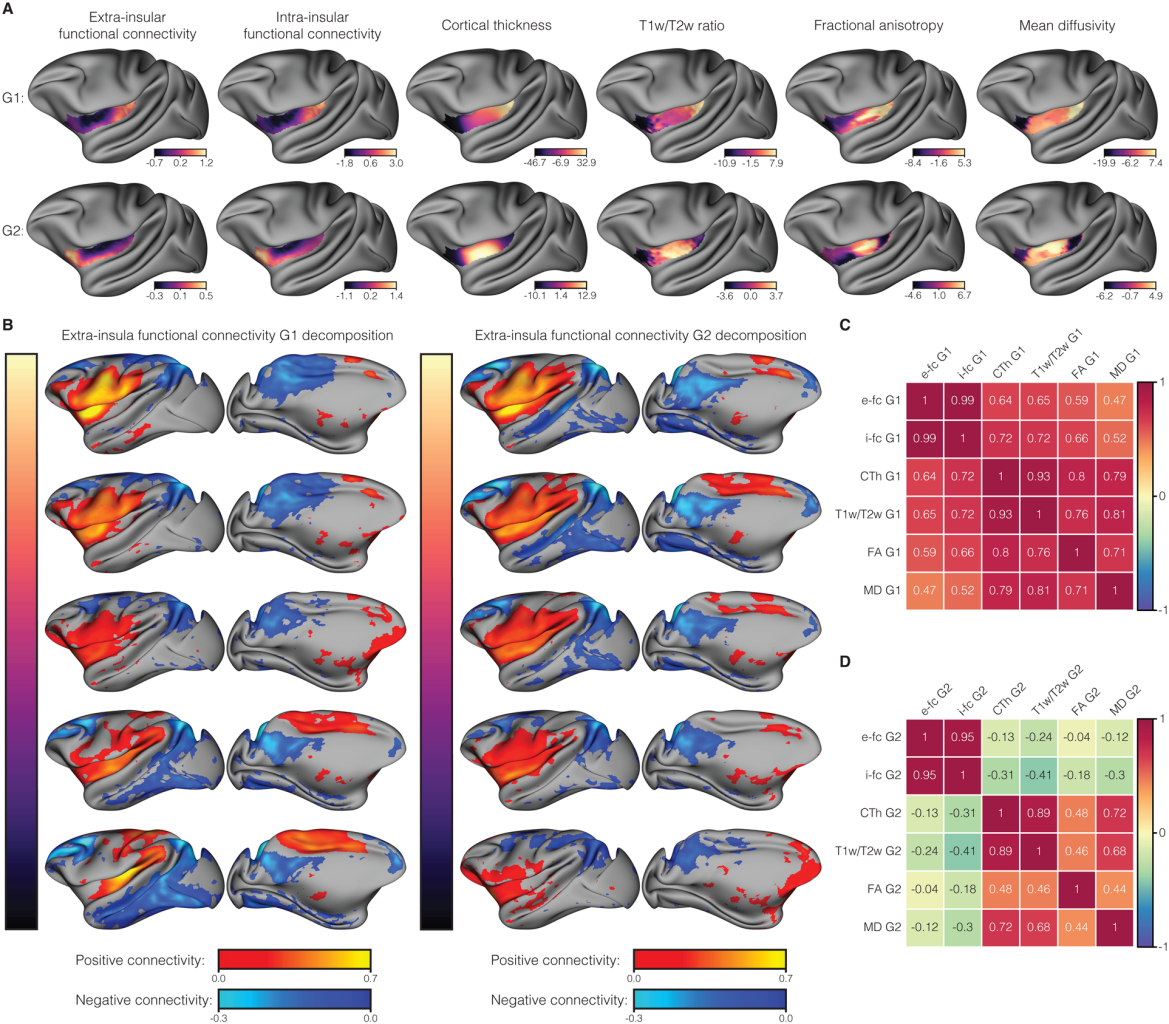
Organizational gradients of functional and structural features of the macaque insula. (A) Gradient maps for all metrics (extrainsular functional connectivity, intrainsular functional connectivity, cortical thickness, T1w/T2w ratio, fractional anisotropy, mean diffusivity) projected onto inflated cortical surfaces. All insula vertices within our region of interest are colored according to their value, and inset color bars reflect the range of values shown on each surface (arbitrary units). Principal gradients (G1) for each metric are shown in the top row and secondary gradients (G2) are shown in the bottom row. (B) Visualization of the results of post hoc characterization of the extrainsular functional connectivity gradients (left: G1; right: G2). Five separate ROIs were generated by discretizing gradients based on voxel-wise gradient values and used in seed-based connectivity analyses. Positive connectivity (i.e., positive correlations in BOLD timeseries) is shown in warm colors and negative connectivity (i.e., negative correlations in BOLD timeseries) is shown in cool colors. An uncorrected statistical threshold of*p*< .05 was used for the generation of surface maps. Maps are shown from a medial and lateral view for each of the five ROIs. The top maps show the connectivity of the seed generated from the highest 20% of the gradient values and the bottom maps show the connectivity of the seed generated from the lowest 20% of the gradient values (as represented by the color bar to the left of each set of surface maps). (C) Correlations between diffusion maps for the principal gradient (G1) derived from each metric (e-fc: extrainsular functional connectivity; i-fc: intrainsular functional connectivity; CTh: cortical thickness; T1w/T2w: T1w/T2w ratio; FA: fractional anisotropy; MD: mean diffusivity). Cells are colored according to the strength of the correlation (deep red: strong positive correlation; deep blue: strong negative correlation). All correlations shown are significant with*p*< .001. (D) Correlations between diffusion maps for the secondary gradient (G2) derived from each metric. Cells are colored as in (C). All correlations were significant with*p*< .001 with the exception of the correlation between e-fc G2 and FA G2 (*p*= .21).

Our functional connectivity gradient analysis evaluated connectivity between each insula voxel and all voxels throughout the rest of the brain (Fig. 4A; Extrainsular functional connectivity). These gradients represent how similarly connected each insula voxel was with the rest of the brain (excluding other insula voxels), including both positive connectivity (i.e., significant positive Pearson correlations in voxel-wise BOLD timeseries) and negative connectivity (i.e., significant negative Pearson correlations in voxel-wise BOLD timeseries). Gradient 1 (Fig. 4A; extrainsular functionality connectivity G1 or e-fc G1) accounted for 43% of the variance and gradient 2 (e-fc G2) accounted for 18% of the variance (seeSupplementary Fig. S1). e-fc G1 revealed an anterior-poster axis of organization, with a marked transition midway through the structure (Fig. 4A), mirroring the primary organizational axis that has been shown in human insula with rs-fMRI data (Veréb et al., 2021) and consistent with theoretical models of insular function in allostatic prediction (Barrett & Simmons, 2015). e-fc G2 revealed a different organizational axis, with posterior to anterior ventral orientation consistent with the forward-downward cascade of information through the insula. This gradient appeared to mirror known cytoarchitectural features of the insula, where the posterior dorsal portion is granular cortex and the anterior-ventral portion is agranular cortex (Evrard, 2019;Evrard et al., 2014;Mesulam & Mufson, 1982a).

To determine the differential connectivity patterns associated with the different gradient values, we discretized the voxel-wise gradient values into five regions of interest (i.e., quintiles from the continuous gradient values) and ran seed-based analyses to determine the patterns of functional connectivity of these ROIs. Changes in functional connectivity from anterior to posterior in e-fc G1 were characterized by shifts in both positive and negative connectivity. Positive connectivity with lateral prefrontal cortex, anterior cingulate cortex, and overlying premotor regions in the anterior insula shifted to positive connectivity with somatosensory and parietal regions in the posterior insula (Fig. 4B). Negative connectivity with much of the temporal cortex in the anterior insula changed to negative connectivity with the parietal cortex in the posterior insula (Fig. 4B).

Changes in functional connectivity from dorsal posterior to ventral anterior in e-fc G2 were also characterized by shifts in both positive and negative connectivity. In the dorsal posterior portion, there was positive connectivity with regions spanning the frontoparietal operculum as well as the cingulate cortex, which shifted to positive connectivity with the orbitofrontal and lateral frontal cortex moving toward the ventral anterior portion. Negative connectivity with the superior temporal sulcus and portions of the posterior parietal cortex also shifted to negative connectivity with parietal, somatosensory, and motor regions in the ventral anterior portion moving from dorsal posterior to ventral anterior (Fig. 4).

When we characterized insula’s functional organization based on functional connectivity between each insula voxel and all other insula voxels (i.e., intrainsular connectivity or i-fc), we found that the first two gradients (i-fc G1 and i-fc G2) explained 50% of the variance (i-fc G1: 34%, i-fc G2: 16%; seeSupplementary Fig. S1) and were highly similar to e-fc G1 and e-fc G2 (Fig. 4), giving further weight to the anterior-posterior and dorsal-ventral axes of organization seen in our clustering results and confirming homologies of fundamental axes of organization between the macaque and human insula.

#### 3.4.2 Structural gradients

We next used the same gradient-based analyses to observe patterns of organization in microstructural features to which*in vivo*imaging is potentially sensitive. Prior*in vivo*work in humans has shown that dMRI metrics sensitive to microstructure (normalized Return to Origin Probability or RTOP) map to heterogeneity in functional connectivity measured by rs-fMRI and a behavioral trait ostensibly dependent on insular function, cognitive control ability (Menon et al., 2020). Similarly, the ratio of T1w/T2w image intensities—a proxy for cortical myelination (Glasser & Van Essen, 2011)—followed a primary anterior-posterior gradient and secondary dorsal-ventral gradient in humans which related to task-based functional activity (Royer et al., 2020). In our monkey sample, we found that the primary gradient (G1) for each cortical thickness, T1w/T2w ratio, FA, and MD also captured an anterior-posterior axis of organization (Fig. 4). G1 accounted for 60% of the variance in cortical thickness, 51% of the variance in T1w/T2w ratio, 29% of the variance in FA, and 44% of the variance in MD (seeSupplementary Fig. S1). These results follow the patterns we saw in e-fc G1 and i-fc G1 as well as those found in the human imaging literature (Cerliani et al., 2012;Menon et al., 2020;Royer et al., 2020;Veréb et al., 2021). Further, postmortem studies in macaques demonstrate a progressive decrease in myelination and cellular density (Evrard et al., 2014;Gallay et al., 2012;Mesulam & Mufson, 1982a) from the posterior to anterior extent. It seems likely that this gradient is driven by the decreasing levels of myelination and cellular density. Interestingly, in comparison with the functional connectivity gradients, those in the diffusion, thickness, and ratio metrics followed a more uniform distribution across the anterior-posterior axis.

Whereas G1 for the structural measures (cortical thickness, Tw/T2w ratio; FA, MD) resembled G1 for the both extra- and intrainsular functional connectivity (Fig. 4A), this was not the case for G2. Across all structural measures, there was a secondary gradient which radiated out from the mid insula in the direction of both the anterior and posterior extremes (Fig. 4). This secondary gradient accounted for 17% of the variance in cortical thickness, 20% of the variance in T1w/T2w ratio, 25% of the variance in FA, and 22% of the variance in MD (seeSupplementary Fig. S1). The precise micro- and/or macroanatomical feature(s) captured by this gradient radiating from the mid-insula is unclear. However, one prior study assessing gradients of structural connectivity in the macaque insula based on*ex vivo*scans of postmortem brains found highly similar primary and secondary gradients, suggesting that this may be a replicable pattern (Cao et al., 2022). In that study, the secondary gradient was significantly positively correlated with raw thickness values and significantly negatively correlated with raw T1w/T2w ratio values, which may suggest that we have captured, with G2, some interaction between myelination, cellular density, and cortical thickness. This possibility is reinforced by the fact that the thickness, T1w/T2w ratio, and diffusion metric gradients show high concordance in our sample.

#### 3.4.3 Convergence of gradients across metrics and modalities

Visual assessment of the diffusion maps across each metric suggested a high degree of similarity between maps for the primary gradient in all metrics and similarity within imaging modality for the secondary gradient (i.e., functional measures appeared similar to each other and structural measures appeared similar to each other but functional and structure measures did not appear similar). To confirm this, we computed the correlation coefficient between the maps for each metric. As expected, there were moderate to strong significant correlations between G1 diffusion maps for all metrics (Fig. 4C). The G1 diffusion maps for the two functional metrics (e-fc and i-fc) were very highly correlated (*r*= 0.99) and there were also strong correlations between each of the structural metrics (ranging from*r*= 0.71 to*r*= 0.93). Moderately strong correlations were observed between functional and structural G1 diffusion maps (*r*= 0.47 to 0.72), suggesting that the primary anterior-posterior organizational axis is supported by both functional and structural features of the insula.

For G2, we again observed very strong positive correlations between the diffusion maps for e-fc and i-fc (*r*= 0.95). For the structural metrics, fractional isotropy was more weakly correlated with each of the other structural metrics (*r*= 0.44 to*r*= 0.48), whereas there were moderately strong correlations between the maps for T1w/T2w ratio and cortical thickness (*r*= 0.89), MD and cortical thickness (*r*= 0.72), and MD and T1w/T2w ratio (*r*= 0.68). We observed weak negative correlations between e-fc/i-fc and each of the structural measures (*r*= -0.04 to*r*= -0.41), suggesting that the secondary axis of organization differed between the structural and functional domains. Further, the G2 diffusion map for FA correlated most weakly with each of the other measures, suggesting that this measure may be sensitive to different features of the insula.

### 3.5 Comparison with tract tracing

We compared the findings from our seed-based connectivity analyses with prior evidence from tract-tracing experiments to understand how findings from*in vivo*imaging compare with histological analyses. Two foundational tract-tracing studies from the early 1980s assessed insula connectivity by injecting the insula with the retrograde tracer horseradish peroxidase (Mufson & Mesulam, 1982) and the anterograde tracer tritiated amino acids (Mesulam & Mufson, 1982b) to evaluate afferent and efferent connectivity, respectively, over the anterior-posterior extent of the structure. We have restyled the figures from these publications for direct comparison with seed-based structural and functional connectivity maps derived from our*in vivo*data shown inFigure 5. Due to the large size of the injections (including the core of the injection and “halo” region in which cells are still likely to uptake and transport neural tracers; seeMufson & Mesulam (1982)), we selected the three seeds from our analyses most closely matching each injection site (based on visual comparison with the figures inMesulam & Mufson (1982b)andMufson & Mesulam (1982)) to directly compare with the tract-tracing connectivity maps. Connectivity maps for each of the three MRI seeds were summed (i.e., including all overlapping and nonoverlapping regions across the seeds) to compare with tract-tracing connectivity.

**Fig. 5.**
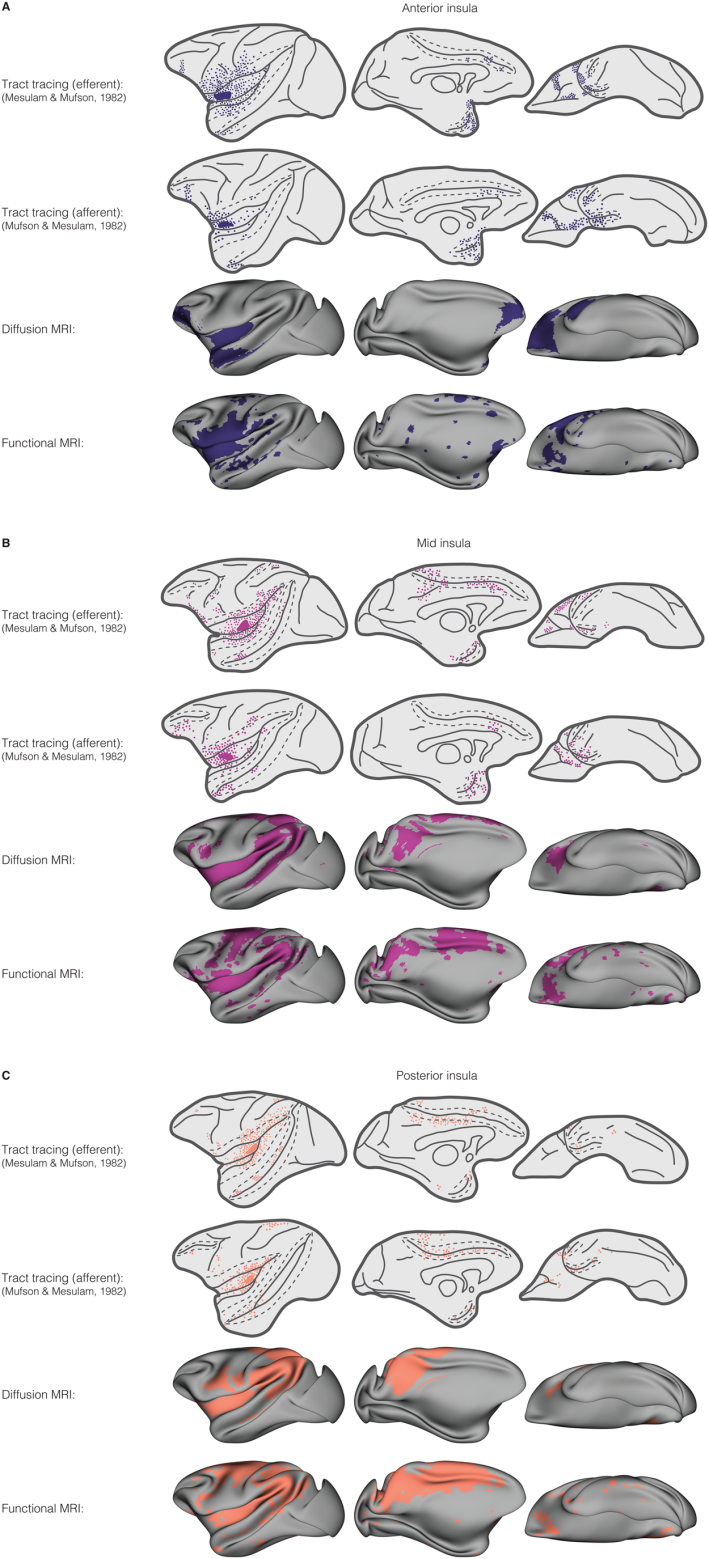
Comparison of the present data with prior tract-tracing evidence. Lateral (left), medial (middle), and ventral (right) surfaces of the brain are shown. Maps display tract-tracing derived efferent connectivity (top row), tract-tracing derived afferent connectivity (second row), imaging-derived structural connectivity (third row), and imaging-derived functional connectivity (last row) for injections/seeds in the (A) anterior (blue), (B) mid (magenta), and (C) posterior (orange) insula. For tracer injection surfaces, the areas between dotted and solid lines represent cortex along the banks of sulci. Similar to inflation of the surfaces for the display of the dMRI and fMRI maps, the lateral sulcus has been opened in the lateral view of the tract-tracing maps to show the inner faces of the operculum, insula, parainsular belt, and supratemporal plane. Labeled regions in the tract-tracing maps are shown with dots and the injection site is shown as a solid-colored region. Anterograde tracer (TAA) injections shown correspond to cases A (anterior insula), B (posterior insula), and C (mid insula) inMufson and Mesulam (1982). Retrograde tracer (HRP) injections correspond to cases A (anterior insula), B (mid insula), and C (posterior insula) inMufson and Mesulam (1982). For the imaging-based connectivity maps, seeds M1, M2, and V3 were used for anterior insula, seeds M6, M7, and M8 were used for mid insula, and seeds D10, M9, and M10 were used for posterior insula (seeSupplementary Fig. S3for the seed locations). Connectivity of the three seeds corresponding to each injection site was summed to create a composite map for each (anterior, mid, posterior) site.

As expected, imaging-derived connectivity did not perfectly recapitulate maps of connectivity derived from tract tracing. However, despite the fact that there were some clear differences across modalities, described in detail below, we observed generally very good correspondence between maps. Notably, imaging maps captured shifts in connectivity from anterior to mid to posterior insula that were present in the tract-tracing data.

#### 3.5.1 Anterior insula connectivity

Anterograde and retrograde tracers in the anterior insula show extensive labeling in frontal regions including granular prefrontal cortex (area 46) and agranular, dysgranular, and granular orbitofrontal cortex (areas 11, 12, and 13) (Mesulam & Mufson, 1982b;Mufson & Mesulam, 1982). We also found evidence for extensive frontal connectivity using both fMRI and dMRI, including connectivity with a stretch of cortex extending anterior from dorsolateral prefrontal cortex (area 46) toward the frontal pole (area 10) and the majority of the orbital surface (areas 10o, 11, 12, 13, and agranular insula). This orbital connectivity extended a bit more anterior toward area 10o on the dMRI map than the tract-tracing connectivity. There was fairly dense efferent connectivity with overlying opercular regions on tract tracing (areas 1, 2, 3a, 3b) which was consistent with our fMRI findings but not our dMRI findings in which we observed fairly minimal opercular connectivity (although we note that dMRI seeds placed slightly more dorsally revealed more extensive opercular connectivity). In line with tract-tracing evidence, both fMRI and dMRI maps showed extensive intrainsular connectivity. We observed cingulate connectivity on both fMRI and dMRI maps, although dMRI connectivity appeared in a field slightly farther anterior (more in line with area 32) than that observed with tract tracing (in area 24). Connectivity with parietal cortex was minimal across tract tracing and imaging, with slightly more connectivity shown on the fMRI, than dMRI, maps. In the temporal lobe, we observed fMRI and dMRI connectivity with the more dorsal aspects of anterior temporopolar regions as well as the anterior tip of the superior temporal sulcus, consistent with efferent and afferent tract-tracing connectivity.

#### 3.5.2 Mid insula

Mid insula connectivity in the tract tracking studies was somewhat similar to anterior insula connectivity, with less extensive frontal connectivity and more extensive connectivity with parietal and more posterior temporal regions (Mesulam & Mufson, 1982b;Mufson & Mesulam, 1982). Just as in the tract-tracing maps, we observed still some frontal connectivity on fMRI and dMRI maps with lateral prefrontal regions (areas 9/46) as well as some more limited connectivity with orbital regions and area 6. Connectivity with area 6 was more extensive on fMRI than observed via tract tracing, although dMRI connectivity between area 6 and mid insula showed very close correspondence to tract tracing. Intrainsular connectivity was again extensive across modalities. This connectivity extended farther posterior, also across modalities, into parietal regions like area PF. In the fMRI data, we observed more extensive connectivity with area 6 which extended farther posterior into area 4 as well, differing from the tract-tracing and dMRI maps. Connectivity with the temporal lobe included more posterior aspects of the superior temporal sulcus on both fMRI and dMRI, mirroring the more posterior temporal connectivity observed from injections in the mid insula. We note that the mid insula injection of the retrograde tracer (horseradish peroxidase) was slightly more anterior than the anterograde tracer (tritiated amino acid) injection and fMRI/dMRI seeds, accounting for the more anterior labeling seen on this map. Cingulate labeling was extensive following anterograde tracer injection into the mid insula, including much of the mid and posterior cingulate gyrus. We observed similar connectivity on fMRI, with more sparse connectivity on dMRI. Across both fMRI and dMRI, we observed midline connectivity which extended farther posterior than observed on tract tracing, including fairly extensive connectivity with posterior parietal and extrastriate visual regions like area 7 m for which there is not tract-tracing evidence.

#### 3.5.3 Posterior insula

In the posterior insula, tract tracing shows evidence of very limited labeling in frontal regions, including lateral frontal and orbital regions as well as area 6 (Mesulam & Mufson, 1982b;Mufson & Mesulam, 1982). We saw similar connectivity patterns on fMRI and dMRI, with much more sparse connectivity across these frontal regions. Connectivity extended farther posterior from area 6 into areas 3a/b, 4, and 5 on fMRI and dMRI, mirroring extensive connectivity with these parietal regions shown via tract tracing. fMRI connectivity, and to a lesser extent dMRI connectivity, between posterior insula and the more dorsal aspects of areas 4 and 6 was more extensive than has been shown via tract tracing. As with the mid insula, we again also observed more extensive midline connectivity with posterior parietal cortex via fMRI and dMRI than has been shown with tract tracing. We also observed extensive connectivity with much of the cingulate cortex on fMRI as shown via tract tracing, with dMRI showing less extensive connectivity in this region. In the temporal lobe, fMRI and dMRI showed connectivity primarily with the more posterior portion of the superior temporal sulcus, with some less extensive connectivity in the more anterior aspects and the inferior temporal gyrus, aligning nicely with tract-tracing evidence.

## 4 Discussion

*In vivo*neuroimaging in macaques has the potential to form a translational bridge between neuroanatomical studies carried out using histological tools in macaques and magnetic resonance imaging (MRI) studies in humans by establishing whether the organization revealed by MRI has a clear basis in ground truth neuroanatomy (e.g., cytoarchitecture, tract tracing), which we do here. We used functional, structural, and diffusion MRI to investigate the structure, organization, and network participation of the macaque insula, harmonizing foundational high-resolution cytoarchitectonic and tract-tracing studies in macaques (Evrard et al., 2014;Gallay et al., 2012;Krockenberger et al., 2023;Mesulam & Mufson, 1982a,1982b;Mufson et al., 1981;Mufson & Mesulam, 1982;Pandya et al., 1981;Vogt & Pandya, 1987) with the extensive neuroimaging literature on insula structure and function in humans (Cauda et al., 2011;Chang et al., 2013;Deen et al., 2011;Kelly et al., 2012;Kleckner et al., 2017;Kurth, Zilles, et al., 2010;Menon et al., 2020;Seeley et al., 2007;Touroutoglou et al., 2012;Uddin et al., 2017;Uddin & Menon, 2009). We partially replicated and expanded on a prior study of ours (Touroutoglou et al., 2016) assessing the functional networks rooted in the macaque anterior insula and used this as a jumping off point for a multimodal characterization of the whole macaque insula. We found evidence for two organizational axes within the insula, the first oriented anterior to posterior, and the second oriented from ventral anterior-to-dorsal posterior. Both axes were apparent across analysis methods (i.e., hierarchical clustering and diffusion map embedding gradients) and imaging modalities (fMRI and dMRI), and were consistent with organizational schemes proposed based on both the results of human imaging studies (Cauda et al., 2011;Chang et al., 2013;Kurth, Zilles, et al., 2010;Menon et al., 2020;Royer et al., 2020;Seeley et al., 2007;Touroutoglou et al., 2012) and macaque anatomical studies (Evrard et al., 2014;Gallay et al., 2012;Krockenberger et al., 2023;Mesulam & Mufson, 1982a,1982b;Mufson et al., 1981;Mufson & Mesulam, 1982;Pandya et al., 1981;Vogt & Pandya, 1987). When we directly compared connectivity maps derived from*in vivo*imaging measures with those derived from tracer injections into the macaque insula (Mesulam & Mufson, 1982b;Mufson & Mesulam, 1982), there were many similarities in connected regions and a few key differences that can guide future study. Our results suggest that the macaque model has high translational potential for future studies of insula structure and function and that insights gleaned from prior—and those that will be gleaned from future—human*in vivo*MRI studies have a strong basis in ground truth anatomical features.

In the present study and our previous report (Touroutoglou et al., 2016), we found evidence that the macaque ventral anterior insula (vAI) was “functionally connected” to (i.e., had correlated BOLD activation with) neural regions important for affective processing (i.e., “limbic” regions), including amygdala, ventral striatum, anterior cingulate cortex (ACC), and orbitofrontal cortex (OFC). Results across the two studies differed to some degree with regard to the connectivity of the dAI seed, perhaps because of methodological differences. In the initial study (Touroutoglou et al., 2016), fMRI data were collected from 4 monkeys while they fixated on a computer screen, while in the present study data were collected from 19 monkeys under light isoflurane anesthesia. Here, we found robust functional connectivity between the dAI seed and affective hubs (similar to the connectivity of the vAI) that were not apparent in our previous report. As a result, in the current data, we did not find evidence that the vAI and dAI networks were statistically dissociable as they were in the previous report (Touroutoglou et al., 2016). We previously suggested that the network rooted in the macaque dAI lacked close homology with the human dAI network due to the absence of connectivity between the dAI and frontal and parietal regions—a key feature of the human dAI network (Touroutoglou et al., 2012). Here, we identified an extensive dAI network that had strong functional connectivity with frontoparietal regions, suggesting that there may be stronger homology than previously reported. These findings are also consistent with a shift that has occurred in the human imaging literature—early studies established that the dAI and vAI networks were dissociable (Deen et al., 2011;Nomi et al., 2016;Touroutoglou et al., 2012), but there is now evidence that there is substantial overlap between these networks and their function (Barrett & Simmons, 2015;Katsumi et al., 2022;Kleckner et al., 2017;Uddin et al., 2014).

It is important to note that although the present work was able to be carried out in a fairly large sample of monkeys because they were under light isoflurane anesthesia, there is some evidence to suggest that isoflurane may have an effect on patterns of resting-state functional connectivity (Giacometti et al., 2022;Lv et al., 2016;Wu et al., 2016). However, the extent to which awake “resting-state” activation in monkeys recapitulates resting-state activity in humans, who can be explicitly told to rest and not otherwise engage in mental processes which may influence activation, is somewhat unclear. Given that we observed fairly consistent patterns of connectivity between fMRI (which may be impacted my mental state) and dMRI (which should not be impacted by mental state) analyses, we have confidence in our reported analyses. Further, several prior reports have compared resting-state functional connectivity in humans with fMRI connectivity in anesthetized macaques under conditions very similar to the present report in order to assess the organization of other neural regions (Hutchison et al., 2012,^2013^,^2015^;Mars et al., 2011;Miranda-Dominguez et al., 2014;Sallet et al., 2013). We have also recently shown that monkeys under conditions similar to those in our present report show significant differences in insula-based network activation under different touch conditions (affective vs. discriminative touch), suggesting that we can capture important features of insula neural activity under light anesthesia (Charbonneau et al., 2024). Still, future work should characterize potential influences of anesthesia on insula-based networks, which may provide insight into insula’s role in conscious awareness more generally.

We found connectivity between the macaque anterior insula and the rest of the brain which was very similar to multiple functional networks of the cerebral cortex associated with insula in humans, including the salience (Seeley, 2019) (or ventral attention (Corbetta & Shulman, 2002)), executive control (Molnar-Szakacs & Uddin, 2022;Trautwein et al., 2016), and default mode (Raichle, 2015) networks. Given the shared hubs across these networks, they are best conceptualized relative to their primary domain-general functionality—the predictive regulation of physiology (i.e., allostasis) through the integration of bodily (i.e., interoceptive) information, with environmental (i.e., exteroceptive) information (Barrett & Satpute, 2013;Gu & FitzGerald, 2014;Katsumi et al., 2022;Kleckner et al., 2017;Sterling, 2012). Tract-tracing studies in monkeys (Augustine, 1996;Friedman et al., 1986;Mesulam & Mufson, 1982b,^1985^) and electrical stimulation and recordings in human patients (Almashaikhi et al., 2014) demonstrate that this information first arrives in the posterior and mid-dorsal portions of the insula before undergoing integration and refinement via intrainsular connections in the mid insula and finally traveling to the anterior portion. Anterior insula ultimately carries out additional integration and processing (Craig, 2011) before outputting to visceromotor control regions and frontal cortical areas, allowing for “higher order” representations of information—particularly in the social and affective domains (Pfeifer & Peake, 2012;Toller et al., 2018).

Both our cluster and gradient analyses of functional connectivity suggested an anterior-posterior organization in the macaque insula, which was further supported by gradient analyses of multiple structural features (cortical thickness, T1w/T2w ratio as a proxy for myelin content, fractional anisotropy, and mean diffusivity). Gradient analyses complement and expand on more traditional seed-based functional connectivity analyses, particularly as there is increasing recognition that low-dimensional representations of neural features have significant implications for domain-general functions like predictive processing (Katsumi et al., 2022). The anterior insula of the macaque was functionally and structurally connected to regions including anterior cingulate, frontal, orbitofrontal, and anterior temporal cortex, and this connectivity changed to include regions such as posterior cingulate, parietal, posterior temporal, somatosensory, and motor cortex proceeding farther posterior. This aligns neatly with human functional connectivity (Cauda et al., 2011;Chang et al., 2013;Deen et al., 2011;Kurth, Zilles, et al., 2010) and tractography (Cerliani et al., 2012;Cloutman et al., 2012;Ghaziri et al., 2017) studies, which have found similar connectivity and organization, as well as with theoretical models of insula’s function in predictive processing (Barrett & Simmons, 2015). When insula was further divided beyond an anterior-posterior split in our hierarchical clustering analysis, we found that a three-part division of the insula fits the data the best—with dorsal-ventral separation of either the anterior or posterior portion—reminiscent of the patterns seen in human fMRI meta-analyses (Chang et al., 2013;Kelly et al., 2012;Kurth, Zilles, et al., 2010). However, it is important to note that as in the human literature (Uddin et al., 2014), in addition to unique connectivity between clusters, there was considerable overlap in the connectivity profiles of different seeds and subregions.

Our imaging analyses provided the important opportunity to rigorously compare connectivity maps derived from*in vivo*imaging with those derived from injection of neural tracers, which detail monosynaptic neural connectivity. Although tract tracing provides evidence of certain interregional connectivity, there are advantages and disadvantages to this methodology compared with*in vivo*imaging. Tracer studies necessitate euthanasia, which makes the application of such methods in humans and longitudinal studies in animals untenable, whereas*in vivo*imaging can be carried out in living humans and nonhuman animal models alike.*In vivo*imaging can also be carried out in far more robust samples, allowing for the identification of individual differences in anatomy. Tract-tracing methods are not easily scaled to assess individual differences and would necessitate nearly identically placed injections and large sample sizes. A single*in vivo*MRI (or cohort of scans) can also be used to seed many different investigations of connectivity, while there is a limit on the number of injections that can reasonably be done in a single monkey. Despite some of the advantages to*in vivo*imaging, it does not allow for an assessment of the direction of connectivity and is likely to identify not only multisynaptic connections, but also spurious connections not grounded in true anatomy. Both of these limitations are likely to account for some of the differences we observed inFigure 5. Direct comparison between tract-tracing connectivity and imaging-derived connectivity, as we have done here, provides the best opportunity to identify potential artifacts that may be present in human imaging data, where direct comparison with tract tracing is not possible. We note, however, that there are many challenges associated with such a comparison. Tract-tracing connectivity maps are often not available at the whole-brain level for comparison with MRI and are not easily registered to a centralized space as MRI data are. There are also some regions like insula and somatosensory cortex for which injection coverage is fairly sparse in the literature (Bezgin et al., 2012). At least one centralized database of tract-tracing results, Collation of Connectivity data on the Macaque brain (CoCoMac;Bakker et al., 2012;Stephan et al., 2001), exists to facilitate whole-brain connectivity tract-tracing comparisons, but is now, in our experience, out of date and missing several studies demonstrating insula connectivity.

The data in the present study were collected from a sample of monkeys who were engaged in a larger noninvasive study on social behavior and were thus returned to their social group following MRI data acquisition (Bliss-Moreau et al., 2021). All of the subjects in our study were male. As such, it remains possible that there may be some sex differences in insula functional and/or structural organization that we were not able to detect here, a limitation of our sample. However, we note that sex differences have not previously been reported in cytoarchitectural studies of the macaque insula (Evrard et al., 2014). Given that monkeys were not euthanized at the conclusion of the present study, we do not have histological data to map*in vivo*findings to anatomical features within subject. However, this work lays a foundation which suggests that further study of the macaque insula making use of subject-matched*in vivo*MRI,*ex vivo*MRI, and histological data has great translational potential. The value of such work is high for basic neuroanatomical insights into brain structure and function, but perhaps even more valuable will be assessments in monkey models of human diseases which compromise the integrity of insula (Christopher et al., 2014;Foundas et al., 1997;Tian et al., 2019) (and other structures) and across the lifespan during early development (Govaert et al., 2004) and very old age (Foundas et al., 1998;Nosheny et al., 2019). Monkeys enable the time-locked collection of*in vivo*and postmortem brain data which is extremely rare in human samples. Such methodology is important because it can provide critical insights into the early stages of disease processes where there is the highest potential for intervention, but when death is an unlikely outcome and so sufficiently high-resolution data are simply not attainable from human subjects. Finally, while the present study is focused on insula and the neural networks in which it is embedded because of the apparently broad relevance of understanding this structure and its function to understanding many different human conditions and diseases, future work can similarly characterize other regions in a directly comparative and translational manner in both healthy and specialized samples to yield a better understanding of the human brain overall.

## Supplementary Material

Supplementary Material

## Data Availability

Data underlying the hierarchical clustering analyses are available on the Open Science Framework athttps://osf.io/xfsgc/. Raw imaging data will be publicly available via the PRIME-DE website (https://fcon_1000.projects.nitrc.org/indi/indiPRIME.html) after all data analyses are complete for this dataset.
