# Supplementary material for: Intrinsic functional and structural network organization in the macaque insula
Source: Imaging Neurosci (Camb). 2024 Aug 14;2:imag-2-00261. doi: 10.1162/imag_a_00261 (PMC12290548; doi:10.1162/imag_a_00261)
Supplement: Supplementary Material [file imag_a_00261-supp.pdf]

Supplementary Materials

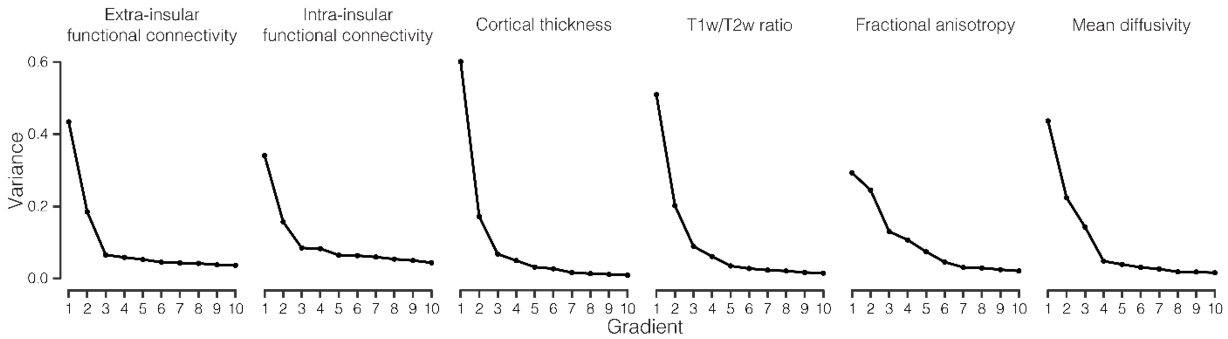

Supplementary Figure 1. Proportion of variance explained by each gradient.

The proportion of variance captured is shown as a function of the gradient number (1-10). For all metrics, more than 50% of the total variance was explained by the primary and secondary gradients. For each metric (extra-insular functional connectivity, intra-insular functional connectivity, cortical thickness, T1w/T2w ratio, fractional anisotropy, mean diffusivity) no gradient beyond the dominant 2 gradients explained more than 15% of the variance, and so the organization of these gradients was not explored in the present analyses.

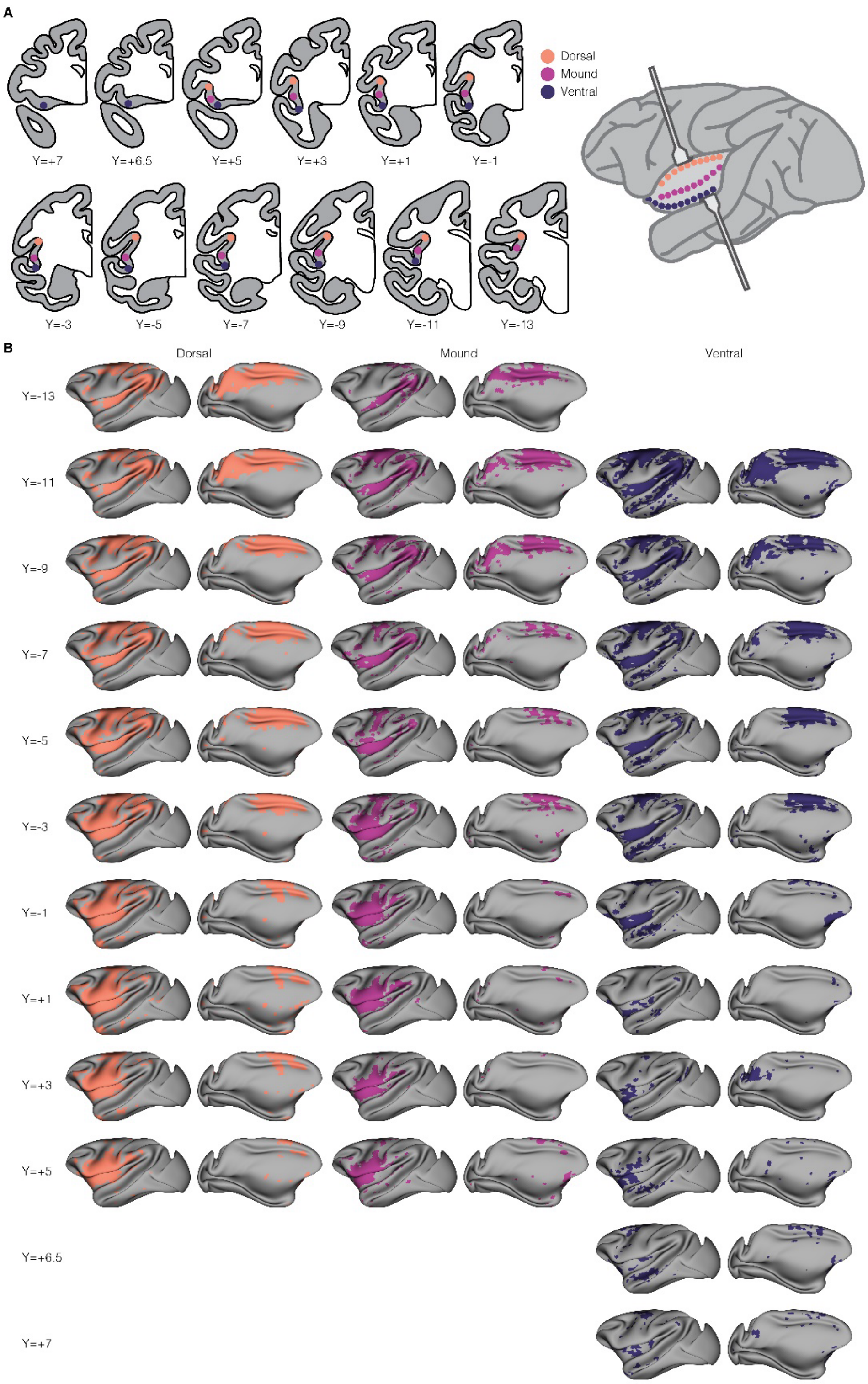

Supplementary Figure 2. Functional connectivity of all insula seeds.

(A) Coronal sections show the placement of 31 seeds tiling the dorsal fundus (orange), mound (magenta), and ventral fundus (blue) of the insula as in Fig. 2A. Each seed was a 2mm diameter sphere placed within the cortical ribbon. A

schematic representation of the macaque brain with the frontoparietal and temporal opercula pulled back to reveal the insula shows approximate placement of the 31 seeds along the surface of the insula. **(B)** Statistical maps showing the topography and magnitude of intrinsic functional connectivity of all insula seeds from (**A**) with the rest of the brain on an inflated surface of the macaque brain. Left hemispheres are shown from a lateral and medial view as seeds were placed in the left hemisphere. The coordinate to the left of each row corresponds to the coordinates shown on the coronal sections in (**A**). Connectivity maps of the dorsal seeds are shown in orange (left), mound seeds in magenta (middle), and ventral seeds in blue (right).

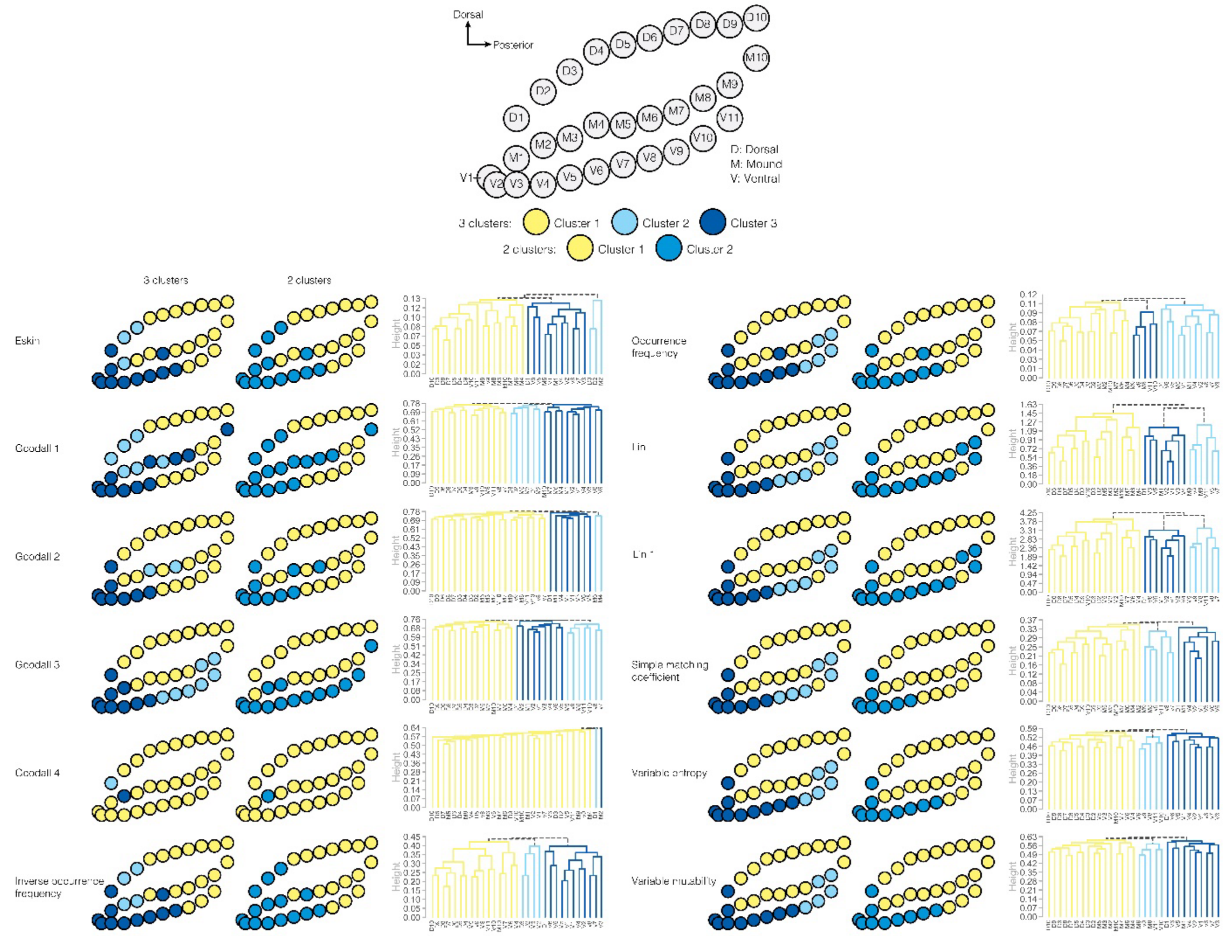

Supplementary Figure 3. All hierarchical clustering solutions.

For each similarity measure two solutions are shown, one with 3 clusters and the other with 2 clusters. Dendrograms corresponding to each solution are shown to the right, colored according to the 3 cluster solutions (but also showing all other possible solutions as clustering was hierarchical). Dendrogram seed labels correspond to the labels in the top schematic (i.e., D1 = dorsal 1, M1 = mound 1, V1 = ventral 1, etc.).

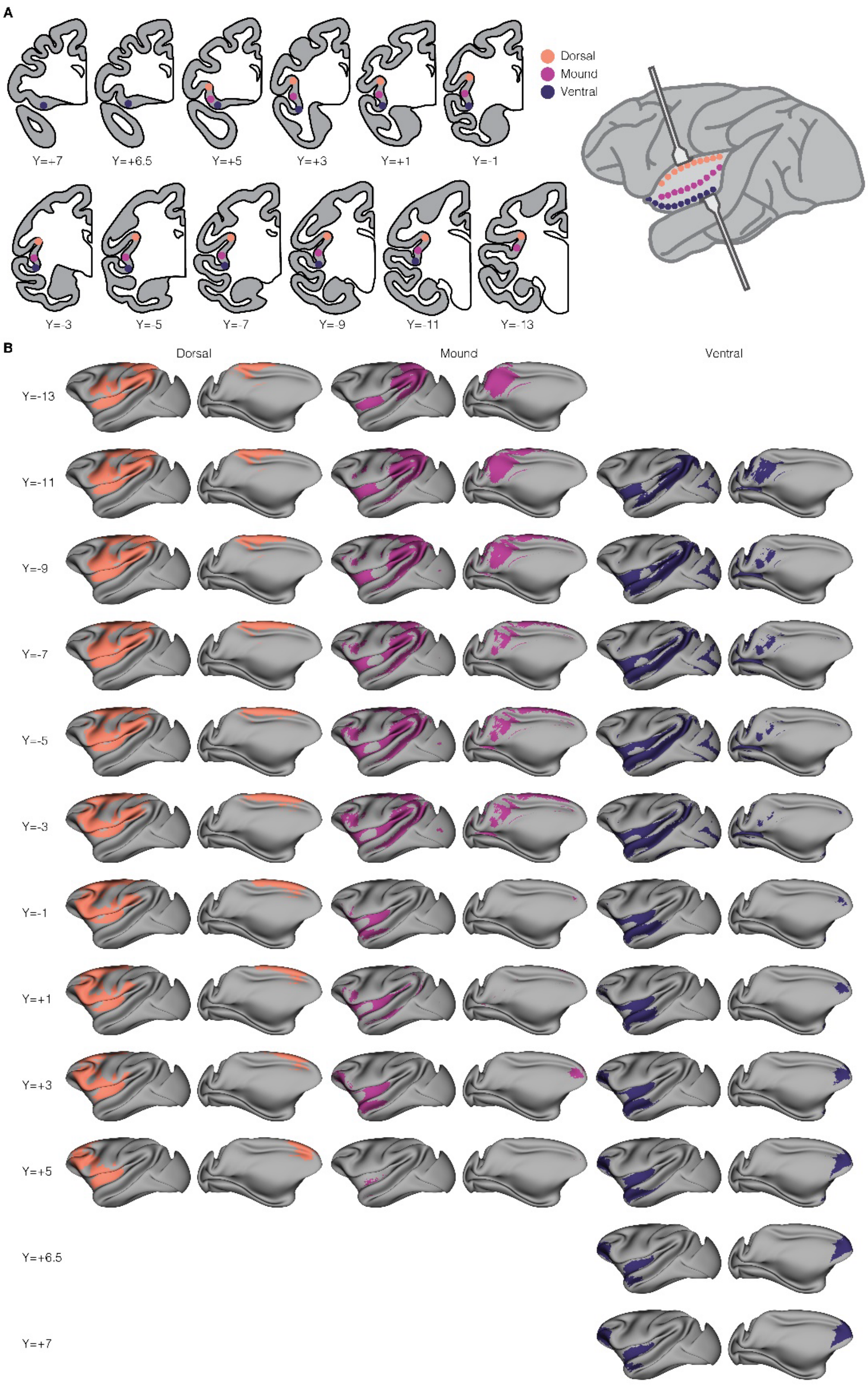

Supplementary Figure 4. Structural connectivity of all insula seeds.

(A) Coronal sections show the placement of 31 seeds tiling the dorsal fundus (orange), mound (magenta), and ventral fundus (blue) of the insula as in Supplementary Fig. 1 and Fig. 2A. **(B)** Maps showing the end points of seed-based

tractography analyses projected onto cortical surfaces for all 31 seeds. Left hemispheres are shown from lateral and medial views. The coordinate to the left of each row corresponds to the seed location in (**A**). Identical seed locations were used as in Supplementary Figure 1. Maps for dorsal seeds are shown in orange (left), mound in magenta (middle), and ventral in blue (right).

Supplementary Table 1. Location of functionally connected clusters identified for the ventral anterior insula seed.

Anatomical area	x	y	z	Z-stat	# of voxels
Ventral anterior insula	17	5	-4	20.8	2337
Intermediate granular insula	-13	9	1	5.34	6
Putamen	-15	-2	-3	9.93	1403
Secondary somatosensory cortex	-24	-12	4	7.51	101
	-24	-19	3	5.44	7
Anterior cingulate cortex					
ACC (24c’)	-1	-5	17	5.71	21
ACC (24c’)	-1	-2	17	5.79	14
Area 25	-2	7.5	-1.5	4.4	
Dorsomedial prefrontal area	6	7	15	5.97	31
Ventromedial prefrontal area	0	18	2	4.96	7
Orbitofrontal cortex	-8	16	4	6.76	55
	3	20	0	5.38	6
Supplementary motor area (F3)	0	6	24	6.85	52
	1	-1	24	6.57	23
Premotor cortex (F2)	5	3	23	7.81	48
	4	-2	24	5.59	7
Parietal opercular area	-23	-16	10	6.16	32
	-25	-19	14	5.73	23
	22	-12	7	5.12	6
Caudal inferior parietal lobule	-20	-21	14	5.44	6
Frontal eye field	18	3	17	5.14	7
Intraparietal sulcus					
AIP	22	-14	13	6.52	31
AIP	-21	-16	13	4.95	10
VIP	7	-37	11	5.29	9
MIP	8	-35	19	5.82	9
MIP	-1	-38	16	5.71	7
LIP	-8	-25	14	5.66	5
Nucleus accumbens	6	4	4	6.01	27
	-5	2	2	5.35	15
	5	7	-1	5.42	11

Superior temporal sulcus					
ventral bank	26	-12	-6	5.73	22
ventral bank	16	3	-18	5.88	12
ventral bank	-27	-8	-10	5.7	11
dorsal bank	-23	-8	-7	6.4	21
dorsal bank	19	2	-9	5.16	9
dorsal bank	-30	-10	-3	5.82	11
temporal pole	18	6	-16	5.03	7
Posterior inferotemporal area	30	-20	-8	5.41	17
Area 46v	16	13	12	5.13	14
	-18	15	15	5.6	8
Area 44 / 45	14	4	14	5.16	6
Area 45	19	4	12	5.19	5
Primary visual cortex	-9	-44	3	6.09	13
Amygdala	13	-2	-12	6.31	12
	-11	-2	-13	5.41	7
Anterior amygdaloid area	-13	1	-8	6.18	8
Caudate	-5	5	5	5.43	10
	8	3	8	4.97	6
V6A	-2	-38	12	5.07	8
Posterior inferotemporal area	-29	-13	-3	5.34	6
Auditory cortex	31	-9	3	6.18	5
	23	-10	0	4.93	5

Supplementary Table 2. Location of functionally connected clusters identified for the dorsal anterior insula seed.

Anatomical area	x	y	z	Z-stat	# of voxels
Dorsal anterior insula	21	2	2	20.2	13590
Ventro-lateral prefrontal area					
Area 46v	16	13	12	6.6	148
12r	-18	14	14	6.75	61
Superior temporal sulcus (STS)					
temporal pole	-17	4	-16	7.09	77
temporal pole	18	5	-16	6.23	45
STS (TEO)	-28	-21	6	4.79	5
STS (TEO)	-24	-20	3	5.12	4
STS (TEa)	23	-5	-11	5.53	4
MST	-19	-27	12	5.46	26
MST	16	-27	10	6.75	23
MST	-17	-19	5	6.21	7
Anterior cingulate cortex					
Area 24c	-5	8	15	6.72	71
Area 24c'	-2	-12	14	4.82	3
Area 25	-2	7.5	-1.5	4.4	
Posterior cingulate cortex					
Area 23c	-6	-18	13	5.34	6
Area 23c	-3	-24	19	5.03	5
Area 23c	5	-30	22	5.43	3
Area 31	1	-25	18	5.49	10
Orbital prefrontal areas					
Area 12m	-14	19	7	5.27	4
Area 11m	20	1	4.94	3	4
Agranular frontal area F1					
	-6	-13	24	5.33	23
	6	-15	25	5.24	4
Somatosensory areas					
Areas 1 and 2	5	-27	21	5.84	20
Areas 3a and 3b	10	-15	23	5.65	20
Primary visual cortex (V1)					

		-12	-44	4	6.02	14
		12	-45	4	7.39	9
		9	-45	4	5.04	3
Secondary visual cortex (V2)						
		5	-39	3	5.95	10
		-5	-38	-1	6.69	9
		7	-44	7	5.3	7
		-3	-42	-1	5.36	6
Amygdala						
		-5	-47	0	5.01	2
		-23	-28	15	4.78	1
		-8	-42	1	4.8	1
hypothalamus		1	-5	-5	5.64	8
Intraparietal sulcus						
	VIP	-7.5	-25	12	4.98	7
	VIP	7.5	-25.5	12.5	6.57	7
	LIP	-13	-14	19	4.83	2
	MIP/LIP/VIP	-8.5	-23	13	7.85	
	MIP/LIP/VIP	8.5	-25	13	6.69	
cd		2	-1	8	5.54	12
Iai		-11	8	-3	5.33	6
Temporo-parietal area (Tpt)		22	-26	10	4.66	2
